# A novel biomacromolecule-predominated hybrid unit: from design, characterization to application

**DOI:** 10.1093/nsr/nwag099

**Published:** 2026-02-14

**Authors:** Ke Hu, Ziying Zhou, Zhaobin Guo, Hongxu Meng, Xuzhi Hu, Jiamin Zhang, Jiayi Zhu, Ruichun Luo, Guoyin Chen, Tingting Yu, Meifang Zhu

**Affiliations:** Department of Biomedical Engineering, School of Biomedical Engineering and Informatics, Nanjing Medical University, Nanjing 211166, China; State Key Laboratory of Advanced Fiber Materials, College of Materials Science and Engineering, Donghua University, Shanghai 201620, China; Institute of Interdisciplinary Integrative Medicine Research, Shanghai University of Traditional Chinese Medicine, Shanghai 201203, China; Zhejiang University-University of Edinburgh Institute, School of Medicine, Zhejiang University, Haining 314400, China; State Key Laboratory of Solid Lubrication, Lanzhou Institute of Chemical Physics, Chinese Academy of Sciences, Lanzhou 730000, China; Department of Biomedical Engineering, School of Biomedical Engineering and Informatics, Nanjing Medical University, Nanjing 211166, China; State Key Laboratory of Advanced Fiber Materials, College of Materials Science and Engineering, Donghua University, Shanghai 201620, China; State Key Laboratory of Advanced Fiber Materials, College of Materials Science and Engineering, Donghua University, Shanghai 201620, China; State Key Laboratory of Advanced Fiber Materials, College of Materials Science and Engineering, Donghua University, Shanghai 201620, China; Department of Medical Genetics, School of Basic Medical Science, Nanjing Medical University, Nanjing 211166, China; State Key Laboratory of Advanced Fiber Materials, College of Materials Science and Engineering, Donghua University, Shanghai 201620, China

**Keywords:** nanohybrid, blob model, mechanical biomimetics, biocatalysis, protein expression technology

## Abstract

Current biomaterial designs struggle with complex clinical and life science demands as single-function approaches are increasingly inadequate, necessitating the systematic integration of four core elements: biosafety, physiological compatibility, biomechanical matching, and biocatalytic function across hierarchical levels. This study addresses the challenge by introducing a novel strategy using natural biomacromolecules to construct microscopic organic-inorganic hybrid units. A comprehensive characterization paradigm employing synchrotron small-angle X-ray scattering, atomic force microscopy coupled with infrared spectroscopy and high-resolution transmission electron microscope was established to reveal emergent hybrid properties. Systematic characterization results demonstrate that the physicochemical properties of these hybrid units more closely resemble polymers than traditional nanomaterials. We introduced the classical polymer blob model to reveal the effects of the hybridization process on the rigidity/flexibility of the polymer chains. Combined characterization results confirmed that the hybrid units possess stable interfaces, bioinspired crosslinking, synergistic high enzyme-like activity with low toxicity, and broad pH tolerance. Multifunctional nanohybrid hydrogel, fabricated with these hybrid units, significantly enhances mammalian cell synthesis of high-quality PD-L1 protein with efficiency improved by nearly an order of magnitude and effectively protects skin organoids from damage caused by exogenous reactive oxygen species. Integrated multi-omics analysis demonstrates that the hydrogel modulates cell–cell/matrix interactions *via* mechano-bioinspiration, boosts endoplasmic reticulum protein processing, and ameliorates hypoxia to enhance mitochondrial respiration (without active oxygen supply), achieving systematic integration of biocompatibility, biomechanics, biocatalysis and physiological environment compatibility. The study also demonstrates the potential of hybrid units in applications such as hydrogel-derived optical fiber fabrication, 3D bio-printing and *in vitro* advanced cell culture models.

## Teaser

A novel organic-inorganic hybrid material through nanohybridization was developed, demonstrating significant potential for applications in wet spinning, 3D printing and organoid culture.

## INTRODUCTION

As biomaterials play an increasingly critical role in clinical therapy, the design philosophy centered on single functionality has proven insufficient to meet complex and growing clinical demands. The performance of biomaterials is far from being determined solely by their core bioactive properties or functions (such as catalytic activity). Their biosafety, biophysical compatibility with biological tissues (e.g. mechanical matching), and biochemical compatibility (e.g. pH) all crucially impact the material’s *in vivo* functionality, long-term stability, and ultimate clinical efficacy [1]. Consequently, the development of biomaterials with multi-dimensional synergistic properties has become a focal point of current research. In the design of multifunctional biomaterials, four indispensable and interconnected core considerations are: biomechanical matching, biocatalytic function, high biosafety, and physiological environment compatibility. High biosafety is the cornerstone, requiring the material and its degradation products to be non-toxic and capable of controlling inflammatory responses [2]. Biomechanical matching is related to functional realization and long-term stability. The material’s mechanical properties must closely resemble those of the target tissue to avoid stress-shielding effects and correctly guide cell behavior [3]. This is currently a hot research topic in simulating the cellular microenvironment *in vitro*. Biocatalytic function endows materials with active intelligence beyond passive support. By integrating enzymes, enzyme mimics, or catalytic nanomaterials, the material can actively catalyze specific reactions, such as: scavenging reactive oxygen species (ROS) at injury sites [4–6]; regulating key signaling molecules [7–9]; sensing environmental changes for intelligent responses [10–13]; or creating favorable microenvironments for tissue engineering. Physiological environment compatibility ensures that the material’s structure remains stable in the complex *in vivo* environment, and its functions can perform steadily under physiological conditions [14]. These four elements act synergistically. the systematic integration of these four points forms the core principle for developing safe, effective, and durable multifunctional biomaterials, determining their ability to successfully fulfill their intended mission in specific biological applications.

**Scheme 1.**
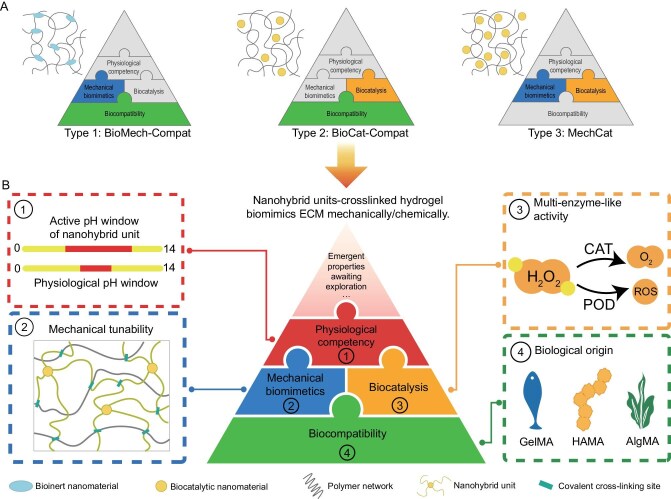
(A) Typical nanohybrid hydrogel network architectures and their biological functions. Type 1: BioMech-Compat (bioinert nanomaterials as crosslinking junctions in polymer networks)—enhances hydrogel network strength while maintaining biocompatibility; Type 2: BioCat-Compat (polymer network composites with low-loading biocatalytic nanomaterials)—provides biocatalytic functionality without compromising biocompatibility; Type 3: MechCat (polymer network composites with high-loading biocatalytic nanomaterials)—achieves dual functionality but typically requires high nanomaterial loading, often leading to reduced biocompatibility. (B) Novel hybrid hydrogel networks constructed with nanohybrid building blocks: BCMP (biocompatibility-biocatalysis-mechanical biomimetics-physiological competency nanohybrid unit)—integrated nanohybrid hydrogel constructed from natural polymer-guided hybrid units as a high-performance biomaterial platform, simultaneously achieving the four core elements: biocompatibility, mechanical biomimetics, biocatalytic function and physiological environment compatibility.

Currently, simple composite materials struggle to meet the aforementioned application requirements. Researchers are attempting to construct biomaterial systems with higher complexity and stability (Scheme 1A). Among these, materials based on nanohybridization strategies have demonstrated outstanding performance across various fields in recent years. In particular, organic-inorganic hybrid hydrogel materials have garnered widespread attention, showing significant application potential in areas like 3D printing [15], brain-computer interfaces [16], and organoid construction [17]. The advantages of existing mainstream nanohybridization strategies centered on inorganic materials for constructing biomimetic materials are as follows: (1) the organic component of the nanohybrid material provides a biomimetic interface and structural support, while the inorganic component imparts intelligent responsiveness and enzyme-like activity [18,19]; (2) hybrid interfaces constructed at the mesoscale enhance the stability of biomimetic materials [20,21]. While these materials have shown promising potential in biomechanics and biosafety, they still face the following shortcomings when confronted with the systematic requirement to simultaneously achieve biomechanical matching, high enzyme-like activity, strong biosafety, and physiological environment alignment: (1) nanohybrid materials built *via* strong interactions (e.g. covalent bonds) can meet mechanical biomimetics requirements, but this often reduces the enzyme-like activity of the inorganic nanomaterials as building blocks. Conversely, increasing the inorganic nanomaterial loading to meet biocatalytic demands increases cellular/tissue toxicity; (2) nanohybrid materials built *via* weak interactions (e.g. hydrogen bonding, electrostatic interactions) can satisfy biocatalytic requirements but fail to guarantee mechanical matching and material stability during service; (3) the pH for optimal enzyme-like activity of commonly used inorganic nanomaterials often poorly matches the pH of the biological environment, leading to a conflict between enzyme-like activity and biosafety. This also limits their application in nanohybrid material construction. This limitation arises from the underutilization of the functions and effects of the organic components within nanohybrid materials (Scheme 1B).

Our research team has long been engaged in the theoretical and practical study of nanohybridization [22]. Inspired by our earlier proposed organic material–mediated organic-inorganic nanohybridization strategy at the mesoscale [23,24], this study pioneers a novel approach: utilizing natural biopolymers from diverse sources to guide the construction of organic-inorganic hybrid units at the microscale, and subsequently building multifunctional biomimetic nanohybrid materials based on these units. The developed hybrid units exhibit the following significant advantages: (1) Interface Engineering Enhances Material Performance: through microscale guidance by natural biomacromolecule derivatives, the constructed hybrid interface endows the material with polymer-like characteristics rather than traditional metallic nanoparticle behavior. This unique interface engineering not only alters the organizational morphology of biomacromolecules but also significantly improves the tunable mechanical properties of the hydrogel system, while preserving the inherent excellent biocompatibility and processability of biomacromolecules; (2) Function-Safety Synergistic Optimization: the hybrid design maintains the complete biocatalytic functionality of the inorganic core while substantially enhancing biosafety through organic-inorganic synergy, addressing the biocompatibility challenges faced by conventional nanocatalytic materials; (3) Emergence of Novel Functional Properties: most notably, while retaining traditional catalase (CAT)-like activity, the hybrid unit achieves dramatically enhanced pH-universal stability of the catalytic activity. This breakthrough characteristic enables stable catalytic efficiency under broad physiological pH fluctuations (pH 4.0–8.0), overcoming the bottleneck of conventional enzyme materials’ susceptibility to inactivation in dynamic microenvironments. This provides unique advantages for biomedical applications in pathological conditions with significant pH heterogeneity, such as inflammatory tissues and tumor microenvironments. Ultimately, this approach achieves the systematic integration of the four core elements for biomimetic materials: biomechanical matching, biocatalytic function, biosafety, and physiological environment compatibility. As a proof-of-concept, the constructed nanohybrid hydrogel based on these hybrid units promotes cellular self-organization into multicellular spheroids by mimicking the physical cellular microenvironment and leverages its physiologically compatible biocatalytic capability to adaptively ameliorate cellular hypoxia without requiring an external oxygen supply. This synergistic effect ultimately enhances the ability of nanohybrid hydrogels as mammalian cell expression systems to synthesize high-quality PD-L1 protein. Synthesis efficiency is increased by nearly an order of magnitude compared to traditional methods. The study also demonstrates the potential of these hybrid units in cutting-edge applications such as hydrogel-derived optical fiber fabrication, combined cell 3D printing and organoid culturing.

## RESULTS AND DISCUSSION

### Blob model analysis of structures and properties of hybrid units

**Figure 1.**
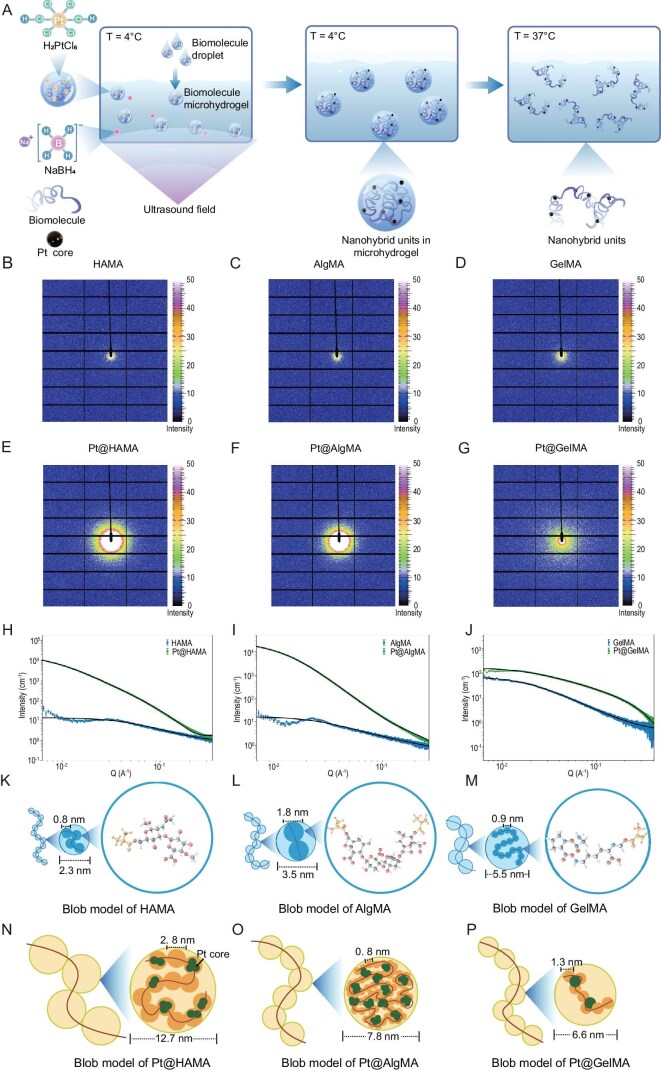
Construction of biomacromolecule-predominated hybrid unit *via* ‘microscopic-scale organically guided inorganic’ preparation strategy. (A) Schematic diagram of the field-assisted synthesis of hybrid units. (B–G) 2D-synchrotron radiation SAXS characterization of the hybrid units and their biomacromolecule precursors. (H–J) Synchrotron radiation SAXS characterization and fitting analysis of the hybrid units and their biomacromolecule precursors. (K–M) Schematic diagram of the blob model for the Pt@HAMA (K), Pt@AlgMA (L) and Pt@GelMA (M) biomacromolecule precursors. (N–P) Schematic diagram of the blob model for the Pt@HAMA (N), Pt@AlgMA (O) and Pt@GelMA (P) hybrid unit.

This study employed a field-assisted controlled phase transition method (Fig. 1A) to innovatively synthesize three types of hybrid units. Chloroplatinic acid served as the platinum source, and three modified biomacromolecules with similar grafted ratio (∼50%) from different sources were selected as ligands: bacterially-derived methacrylated hyaluronic acid (HAMA), plant-derived methacrylated sodium alginate (AlgMA), and animal-derived methacrylated gelatin (GelMA). Notably, due to the substantial chain length imparted by the high molecular weight of the biomacromolecules and the low metal content ratio, the properties of these hybrid units resemble polymers more than nanoparticles. X-ray photoelectron spectroscopy (XPS), thermogravimetric analysis (TGA), Fourier transform infrared spectroscopy (FTIR) and nuclear magnetic resonance (NMR) analyses confirmed that these hybrid units mainly retain the composition, thermal stability, and characteristic functional groups of their respective natural polymers (Figs S1–S4). Additionally, the good redispersion behavior of the freeze-dried samples further indicates the stable interfacial properties of the hybrid units (Figs S5–S7).

To investigate the fundamental structure of these novel hybrid units, we performed synchrotron radiation small-angle X-ray scattering (SAXS) on both the hybrid units and their polymeric ligands. The 2D scattering patterns (Fig. 1B–G, Fig. S8) exhibit uniform concentric rings, indicating isotropic scattering with no preferred orientation. Therefore, we analyzed the samples using the blob model [25] to describe their structural characteristics. In this model, a polymer chain is viewed as a string of blobs (analogous to a ‘pearl necklace’ model [26]). Within each blob, monomers move independently, while the statistical behavior of the entire chain is determined by the arrangement of the blobs. We assumed each blob has a size $\xi $, containing monomers of size *l*. The fractal dimension ${d}_f$ (ranging from 1 to 3) dictates the packing of monomers within the blob—a higher ${d}_f$ indicates denser monomer packing. Fitting the SAXS data obtained with synchrotron radiation source provided the above parameters (Table S1). The blob size $\xi $ and the number of monomers per blob (${N}_{\textit{blob}}$) obey a fractal scaling law. Using Equation 1 [25], we calculated the number of monomers contained within each blob


(1) \begin{eqnarray*}
{N}_{\textit{blob}} \approx \ {\left( {\frac{\xi }{l}} \right)}^{{d}_f}.
\end{eqnarray*}

However, SAXS characterization reflects the electron density fluctuations of the entire system. The dispersed and minute platinum metal cores may be obscured by the strong signal from the abundant polymer chains. Therefore, we utilized transmission electron microscope (TEM) to characterize the metal cores. Through combined analysis of SAXS (Fig. 1H–J) and TEM (Figs S5–S7) data, we constructed schematic diagrams illustrating the blob model for the biomacromolecules (Fig. 1K–M) and the hybrid units (Fig. 1N–P). The blob size $\xi $ serves as a measure of polymer chain flexibility: a smaller $\xi $ indicates higher flexibility and greater ease of chain bending [27]. The flexibility order for the three biomacromolecules was HAMA > AlgMA > GelMA. Although, based on monomer structure, HAMA and AlgMA (polysaccharides with sugar ring backbones) might be expected to exhibit lower flexibility compared to GelMA (with an amino acid backbone), our experimental results showed that GelMA’s amino acid chains had larger blob sizes in solution, containing more monomers. This apparent contradiction can be explained by polymer chain–environment interactions. HAMA and AlgMA possess numerous hydroxyl and carboxyl groups, leading to stronger hydration and higher negative charge. The thick hydration layer and electrostatic repulsion cause chain extension, resulting in increased apparent flexibility. After synthesizing the hybrid units, the $\xi $ values changed significantly. The flexibility order of the hybrid units became Pt@HAMA < Pt@AlgMA < @Pt@GelMA, indicating that metal core formation substantially impacted the flexibility of the polymer chain. Both Pt@HAMA and Pt@AlgMA showed an increase in ${d}_f$, signifying denser monomer packing within the blobs. Notably, Pt@AlgMA reached a ${d}_f$ of 2.91, approaching a collapsed chain conformation where monomers are densely packed. This may be due to Pt nuclei forming between AlgMA chains, acting as inter-chain crosslinking points. Conversely, Pt@GelMA exhibited a decrease in ${d}_f$, demonstrating the diverse effects metal cores can have on local chain flexibility. We performed small-angle neutron scattering (SANS) on Pt@HAMA (Fig. S9); fitting of the results confirmed a Pt core size of 1.5 nm, which is consistent with the TEM results.

**Figure 2.**
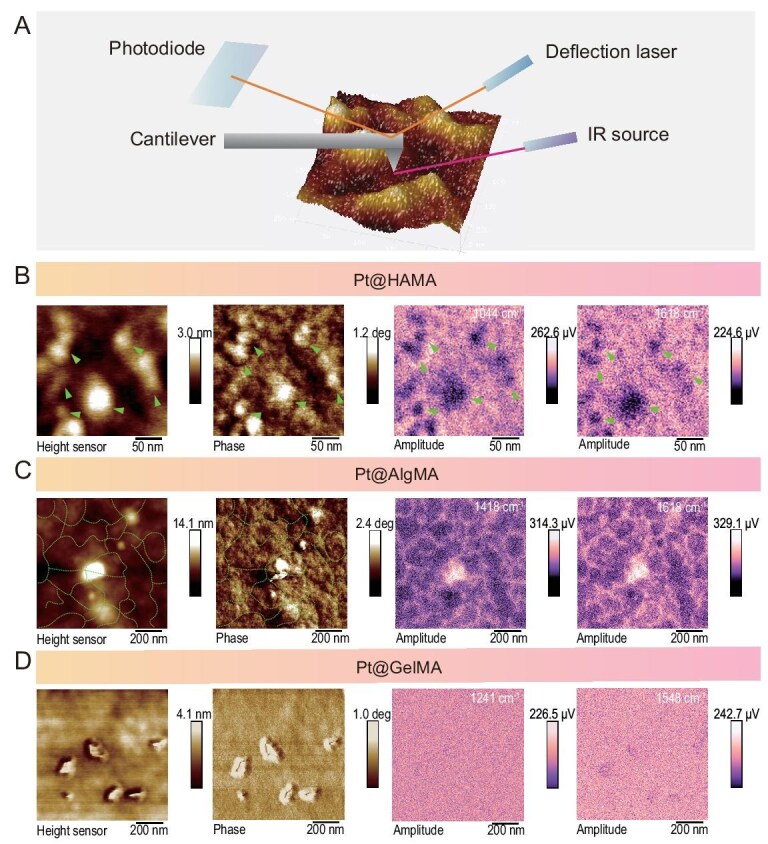
AFM-IR reveals that the microstructure of the hybrid units aligns with the constructed blob model. (A) Schematic diagram of the AFM-IR characterization method. AFM characterization results for the three hybrid units Pt@HAMA (B), Pt@AlgMA (C) and Pt@GelMA (D): height map; phase map; IR-AFM mapping images at different characteristic peaks. Scale Bar: 50 nm for (B) and 200 nm for (C) and (D).

Subsequently, we employed atomic force microscopy coupled with infrared spectroscopy (IR-AFM) to conduct non-destructive characterization of the hybrid units. By scanning the samples at characteristic IR wavelengths, we determined the spatial distribution of the polymeric material within the area (Fig. 2A). In IR-AFM mapping images, lighter colors indicate stronger IR signals, corresponding to regions of higher polymer concentration. For Pt@HAMA (Fig. 2B): the highest regions in the height map correspond to the darkest areas in the IR map. The maximum height of ∼3.0 nm is close to the Pt core size determined by neutron scattering. We attribute these dark regions to hybrid units containing the metal core, where the presence of the core attenuates the polymer IR signal. For Pt@AlgMA (Fig. 2C): in contrast to Pt@HAMA, the lighter areas in the IR map exhibit a distinct network-like pattern. We measured an average branch width of ∼20 nm for this network, which is close to the blob size ($\xi $). We attribute this network morphology observed upon drying to the stacking arrangement of blobs, where the dense packing of monomers within each blob results in stronger IR signals. For Pt@GelMA (Fig. 2D): possessing the highest flexibility and with Pt cores maintaining uniform distribution even after drying, the IR signal distribution is significantly more uniform.

Through detailed characterization of the hybrid unit structure, we can predict their performance in mechanical biomimetics and catalytic function. As crosslinking points, higher rigidity should contribute more significantly to enhancing the mechanical properties of hydrogels. The greater the increase in $\xi $ value from the biomacromolecule to the hybrid unit, the more substantial its role in improving the hydrogel’s mechanical strength. Therefore, Pt@HAMA—exhibiting the largest increase in $\xi $—should demonstrate the most pronounced mechanical reinforcement effect, followed by Pt@AlgMA and then Pt@GelMA. Regarding catalytic function, enzymatic reaction rates depend on multiple factors such as the catalytic center [28], substrate binding capacity [29], and protein surface rigidity [30]. As enzyme mimics, the catalytic efficiency of hybrid units is similarly influenced. Since the three hybrid units possess monodisperse metal cores of comparable size and type, their catalytic efficiency should be predominantly governed by polymer chain conformation. Pt@AlgMA exhibits overly dense monomer packing within blobs, which may impede substrate diffusion to the catalytic center. Pt@GelMA chains are excessively flexible, leading to reduced effective collisions between the substrate and the catalytic active center [31]. Pt@HAMA, with its moderate fractal dimension, should exhibit optimal catalytic efficiency [32]. We conducted mechanical tests on hydrogels constructed with these hybrid units using bio-nanoindentation. Results show that when copolymerized with GelMA (3% w/w), nanohybrid hydrogels built with Pt@HAMA units exhibited a Young’s modulus of 319.77 ± 29.86 Pa—∼6.7-fold higher than hydrogels built with the same concentration of HAMA alone (47.62 ± 3.67 Pa) (Fig. S10A and D). Nanohybrid hydrogels built with Pt@AlgMA units had a Young’s modulus of 195.4 ± 23.68 Pa—only ∼2.1-fold higher than hydrogels built with AlgMA alone (92.70 ± 35.61 Pa) (Fig. S10B and E). Nanohybrid hydrogels built with Pt@GelMA units showed a Young’s modulus of 15.3 ± 0.9 Pa—a minimal ∼1.5-fold increase compared to hydrogels built with GelMA alone (10.09 ± 0.514 Pa) (Fig. S10C and F). These results align perfectly with the earlier $\xi $ analysis of hybrid unit blob size. They fully demonstrate the broad tunability and diversity of hybrid units in regulating nanohybrid hydrogel mechanical properties as well as the feasibility of Pt@HAMA as a building unit for nanohybrid hydrogels to mimic the mechanical properties of the cellular microenvironment.

Simultaneously, we performed catalytic tests on hydrogels crosslinked with the three hybrid units. The CAT-like enzyme activity of the hybrid hydrogels was assessed by monitoring the rate of oxygen bubble generation from hydrogen peroxide decomposition (Fig. S10G and Video S1). The POD-like enzyme activity was evaluated by catalyzing the oxidation of 3,3′,5,5′-tetramethylbenzidine (TMB) to form blue oxidized TMB (oxTMB) (Fig. S10J and Video S2). Results demonstrate that Pt@HAMA exhibits the highest catalytic activity for both CAT-like (Fig. S10G and Video S1) and POD-like (Fig. S10J and Video S2) reactions, consistent with our predictions. We further conducted detailed studies on the enzyme-like activities of the hybrid units in aqueous solution. Dissolved oxygen curves (Fig. S10H) reveal that Pt@HAMA catalyzes H_2_O_2_ decomposition to O_2_ at a rate significantly higher than the other two units. Initial reaction rates for CAT-like activity were measured across different pH values (Fig. S10I). Unlike traditional materials, the hybrid units exhibit broader pH adaptability, maintaining substantial catalytic activity even under acidic conditions (pH = 4). Pt@HAMA consistently showed the highest initial reaction rate at every pH tested. pH dependence of POD-like activity (Fig. S10K) shows strong catalytic capability within the pH 3–5 range. Reaction rates increased linearly with platinum concentration, satisfying the conditions for applying Michaelis–Menten kinetics. To quantify catalytic efficiency, steady-state kinetic experiments were performed (Fig. S10L–N). Kinetic parameters for the hybrid units are listed in Table S2. For the TMB substrate, fitting to the Michaelis–Menten equation yielded a maximum initial velocity (*Vmax*) of 57 µM·min⁻^1^ for Pt@HAMA—6.7 times that of Pt@AlgMA and 14.3 times that of Pt@GelMA. For the H_2_O_2_ substrate, Pt@HAMA achieved a *Vmax* of 82.86 µM·min⁻^1^—7.9 times that of Pt@AlgMA and 13.4 times that of Pt@GelMA. These results highlight Pt@HAMA’s strong potential for biocatalytic applications.

### Biofabrication applications of polymerizable hybrid units

**Figure 3.**
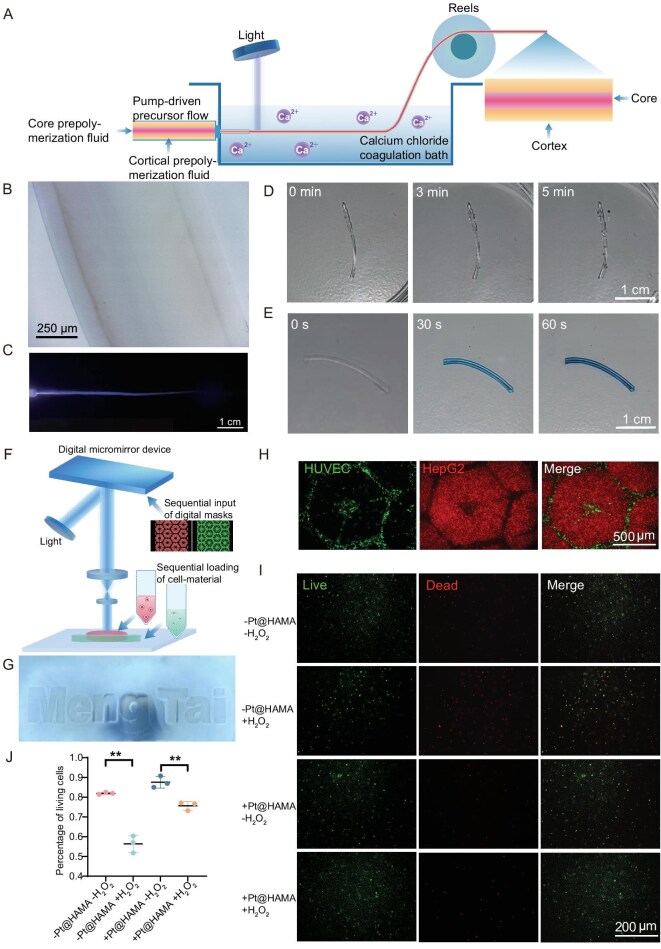
Hybrid units retain polymerizability for optical fiber spinning and bio-additive manufacturing. (A) Schematic of nanohybrid hydrogel optical fiber spinning using Pt@HAMA. (B) Microscopy image of a nanohybrid hydrogel optical fiber. (C) Light-guiding properties of a nanohybrid hydrogel optical fiber. (D) CAT-like enzyme catalytic reaction of a nanohybrid hydrogel optical fiber. (E) POD-like catalytic reaction of a nanohybrid hydrogel optical fiber. (F) Schematic of Pt@HAMA hybrid units for biological 3D printing. (G) 3D-printed nanohybrid hydrogel displaying the research group’s logo (‘MengTai’). (H) 3D-bioprinted hepatic lobule unit co-cultured with multiple cell types: endothelial cells (HUVEC), hepatocytes (HepG2) and merged view. Scale Bar: 500 µm. (I and J) Live/dead 3D-printed hydrogel containing hepatocytes (HepG2) without hybrid units/without H_2_O_2_, without hybrid units/with H_2_O_2_, with hybrid units/without H_2_O_2_, with hybrid units/with H_2_O_2_ (from top to bottom. Scale bar: 200 µm), and associated statistics (J). **p < 0.01, each point represents one biological replicate.

Given Pt@HAMA’s exceptional mechanical reinforcement capability and biocatalytic activity, we synthesized three Pt@HAMA variants with different polymer ratios (Figs S11–S14) and tested their catalytic performance. The group with the strongest catalytic capability was selected for subsequent applications (Figs S15–S19). Following our group’s established protocol [33], hybrid units were incorporated into the shell layer for hydrogel optical fiber spinning (Fig. 3A). The resulting fibers exhibited a distinct core-shell structure (Fig. 3B), excellent light-guiding properties (Fig. 3C), significant CAT-like enzyme catalytic activity (Fig. 3D), and POD-like enzyme catalytic activity (Fig. 3E). This confirms the spinnability of the hybrid units and suggests promising applications for hybrid hydrogel fibers in antioxidant, antibacterial, and other biological scenarios. Building on this, we validated the processability and biocompatibility of hybrid units *via* photopolymerization-based 3D printing (Fig. 3F). Blended with HAMA (2%), they were printed into our group’s logo (‘MengTai’). As shown in Fig. 3G, the logo is clear and structurally intact, demonstrating excellent processability and suitability as a 3D printing matrix material. Post-printing, the hybrid units retained their biocatalytic function, rapidly converting hydrogen peroxide into oxygen (Video S3). This establishes the preliminary suitability of hybrid hydrogels as bioinks for 3D printing artificial organ constructs. Hepatocytes (HepG2, pre-labeled with red fluorescent dye) were printed into hexagonal hepatic lobule structures (side length: 500 µm, height: 300 µm). Uncross-linked bioink and cells were washed away. Endothelial cells (HUVEC, pre-labeled with green fluorescent dye) suspended in bioink were printed into channel structures (side length: 50 µm, height: 300 µm) around the lobules. As shown in Fig. 3H–J: hepatic lobules maintained clear morphology and boundaries (Fig. 3I). Endothelial channels remained structurally intact (Fig. 3H). This confirms the potential of these hybrid units as matrix materials for 3D bioprinting artificial liver constructs. Based on these findings, viability staining was performed on the hexagonal hepatic lobule structures printed solely with hepatocytes. It was observed that after 2 days of culture, the cell viability in the group containing hybrid units was recorded at 87.6%, which was slightly higher than the group without hybrid units (81.9%) (Fig. 3H and I). This result confirmed that nanohybrid hydrogels can be utilized as matrix materials for live-cell 3D printing without causing significant damage to cells. Furthermore, the protective effect of hybrid units on hepatocytes under oxidative stress conditions was evaluated. It was found that after 24 hours of treatment with 500 µM H_2_O_2_, the cell viability in gels without hybrid units declined to 56.4%, significantly lower than that in the group containing hybrid units (75.6%). These results demonstrated the protective role of hybrid units for cells, particularly under oxidative stress conditions.

### Mechanism of hybrid units as biomaterial building blocks

**Figure 4.**
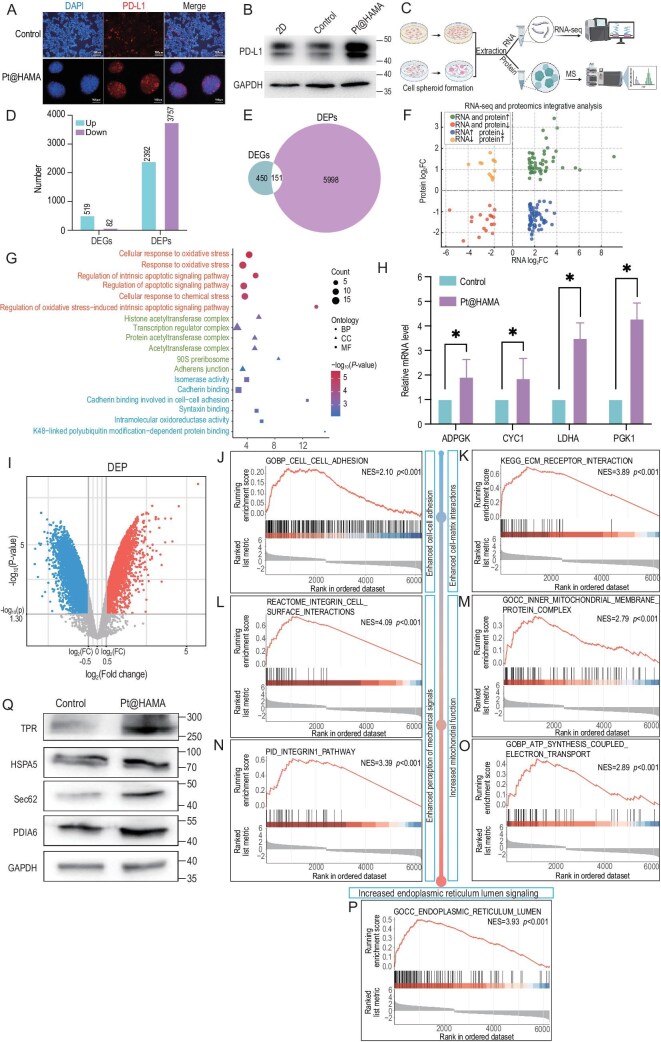
Pt@HAMA nanohybrid hydrogel promotes multicellular spheroid formation and enhances PD-L1 expression in 293T-PDL1(h) cells. (A) Immunofluorescence staining of 293T-PDL1(h) cells cultured in Pt@HAMA hydrogel, showing the formation of compact multicellular spheroids and elevated PD-L1 expression compared to 2D culture. Red: PD-L1; blue: DAPI. Scale bar: 100 μm. (B) Western blot analysis confirming increased PD-L1 protein levels in cells cultured in Pt@HAMA hydrogel compared to 2D conditions. (C) Schematic workflow of combined RNA-seq and proteomic profiling performed on 293T-PDL1(h) cells cultured under 2D and Pt@HAMA conditions for 14 days (*N* = 3 for each group). (D) Quantification of differentially expressed genes (DEGs) and proteins (DEPs) from transcriptomic and proteomic analyses, indicating stronger changes at the protein level. (E) Venn diagram illustrating the overlap between DEGs and DEPs. (F) Four-quadrant scatter plot displaying the concordance of RNA and protein expression changes among intersecting genes identified in panel (E). (G) GO enrichment analysis of overlapping genes, showing biological process (BP), molecular function (MF), and cellular component (CC) terms enriched in oxidative stress response, apoptosis regulation, and cadherin-mediated cell–cell adhesion. (H) RT-qPCR validation of selected metabolism-related genes (e.g. *ADPGK, CYC1, LDHA, PGK1*) that were upregulated at both RNA and protein levels in hydrogel-cultured 293T-PDL1(h) spheroids, suggesting metabolic reprogramming under 3D conditions (*N* = 3 for each group). (I) Volcano plot of differentially expressed proteins in 293T-PDL1(h) cells cultured in control hydrogels vs nanohybrid hydrogels. (J–M) GSEA analysis revealing significant upregulation of pathways related to cell–cell interaction (J), ECM–receptor interaction (K), integrin–cell surface interaction (L), and integrin-1 pathway (M) in hydrogel-cultured spheroids. (N–P) GSEA analysis indicating enhanced mitochondrial function and protein processing, including inner mitochondrial membrane protein complexes (N), ATP synthesis coupled electron transport (O), and ER lumen-related genes (P). (Q) Western blot analysis confirming elevated expression of ER protein processing regulators in nanohybrid hydrogel-cultured 293T-PDL1(h) cells.

Building upon our prior research findings, we propose that Pt@HAMA hybrid units hold significant potential for constructing advanced *in vitro* cell culture models. To evaluate the capability of nanohybrid hydrogels built with Pt@HAMA units as cell culture platforms, we encapsulated 293T-PDL1(h) cells—a mammalian cell line stably transfected with PD-L1—with both Pt@HAMA nanohybrid hydrogels and control hydrogels prepared with HAMA instead of Pt@HAMA [both co-polymerized with GelMA (3% w/w)]. Results revealed that 293T-PDL1(h) cells formed compact multicellular spheroids within the nanohybrid hydrogels (Fig. 4A), whereas cells in control hydrogels exhibited morphology similar to conventional 2D cultures. Both immunofluorescence and Western blot analyses confirmed significantly higher PD-L1 protein expression in spheroids cultured in nanohybrid hydrogels compared to controls (Fig. 4A and B). To gain deeper insights, we collected 293T-PDL1(h) cells cultured for 14 days in control and nanohybrid hydrogels for RNA-seq and proteomic profiling (Fig. 4C). Data indicated that the nanohybrid hydrogel primarily altered cellular expression profiles at the protein level (Fig. 4D), suggesting that nanohybrid hydrogel culture may modulate the proteome of 293T-PDL1(h) cells predominantly through post-transcriptional regulation. Integrative analysis of differentially expressed genes (DEGs) from RNA-seq and proteomics identified 151 genes altered at both RNA and protein levels (Fig. 4E). Approximately 30% exhibited consistent upregulation across both levels (Fig. 4F). Gene ontology (GO) enrichment analysis of these intersecting genes indicated significant involvement in oxidative stress responses and apoptosis regulation. Moreover, these genes were also enriched in cadherin-mediated cell–cell adhesion pathways, potentially contributing to the observed spheroid formation in the 3D culture system (Fig. 4G). To validate these findings, we conducted RT-qPCR on a subset of genes that were upregulated at both transcript and protein levels. Key metabolic regulators—including *ADPGK, CYC1, LDHA*, and *PGK1*—exhibited elevated expression in spheroids cultured within the hydrogel, suggesting that this platform may also induce metabolic reprogramming. Together, these results highlight the ability of Pt@HAMA to promote spheroid formation, enhance PD-L1 expression, and modulate key transcriptional and post-transcriptional pathways in 293T-PDL1(h) cells (Fig. 4H).

To further investigate the mechanism by which nanohybrid hydrogel culture promotes multicellular spheroid formation and enhances PD-L1 expression in 293T-PDL1(h) cells, we conducted comparative proteomic profiling of spheroids cultured in nanohybrid hydrogels versus control hydrogels. Mass spectrometry identified >5000 proteins with significantly altered expression under nanohybrid hydrogel culture (Fig. 4I). Differentially expressed proteins were primarily enriched in metabolic pathways, cell cycle regulation, and endoplasmic reticulum (ER) protein processing (Fig. S20). Further Gene set enrichment analysis (GSEA) further demonstrated that spheroids formed within the Pt@HAMA hydrogel exhibited strong upregulation of gene sets related to cell–cell adhesion (Fig. 4J) and extracellular matrix (ECM)—receptor interactions (Fig. 4K), particularly integrin-mediated signaling pathways (Fig. 4L and N). These findings suggest that the hydrogel matrix provides a biophysical microenvironment that fosters both intercellular and cell–ECM interactions, thereby contributing to the formation of densely packed, three-dimensional spheroids. In addition, genes encoding mitochondrial inner membrane protein complexes and components of the ATP-generating electron transport chain were significantly upregulated in nanohybrid hydrogel-cultured spheroids (Fig. 4M, O, and P). This implies that oxygen released from the Pt-based nanostructures may freely diffuse into the spheroids, improving mitochondrial respiration and enhancing cellular energy metabolism. Consistent with previous findings that Pt@HAMA hydrogel culture promotes PD-L1 production, we investigated whether increased biosynthetic capacity contributed to this effect. We observed significant enrichment of ER lumen-related gene sets, indicative of enhanced protein folding and processing capacity in spheroids. Western blot analysis confirmed upregulation of key ER chaperones and folding regulators, including *TPR, HSPA5, Sec61*, and *PDIA6* [34–36] (Fig. 4Q), suggesting that the nanohybrid hydrogel microenvironment supports efficient post-translational maturation of nascent proteins. Collectively, these data reveal that the Pt@HAMA nanohybrid hydrogel facilitates the formation of compact multicellular spheroids of 293T-PDL1(h) cells, enhances the ER’s capacity to fold and process nascent proteins, and supplies oxygen through the hybrid units, ultimately achieving efficient target protein expression. Protein–protein interaction (PPI) analysis was performed on pathways upregulated in multicellular spheroids, including those associated with the endoplasmic reticulum (ER), cell–cell adhesion, ECM organization, and mitochondrial function (Fig. S21). The results revealed strong interactions among the pathways related to cell–cell adhesion, ECM structure, and the ER lumen, suggesting that the architecture of multicellular spheroids may significantly influence ER functionality. Moreover, interactions of varying intensities were observed between the cell–cell adhesion pathway and the other four pathways, indicating that the establishment of a 3D structure may serve as a fundamental basis for subsequent biological responses. These findings further underscore that the biomimetic mechanical microenvironment provided by the composite hydrogel matrix exerts a pronounced impact on cellular function.

**Figure 5.**
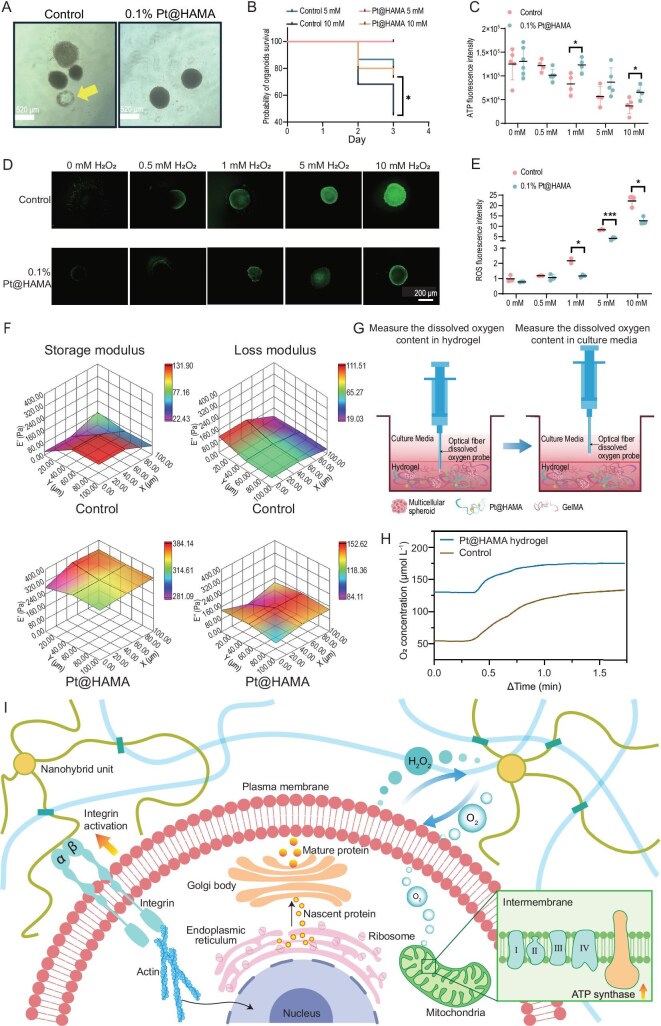
Nanohybrid hydrogel for antioxidant protection of skin organoids and mechanistic analysis of interaction between nanohybrid hydrogels and cells. (A) Representative images of skin organoids cultured in different hydrogels after H_2_O_2_ stimulation. Disintegrated organoid spheroids are marked with yellow arrows. Scale bars: 520 μm. (B) Survival curves of skin organoids cultured under different gel conditions after treatment with 5 mM and 10 mM H_2_O_2_ (*n* = 20). Significance was analyzed by Student’s *t*-test. (C) ATP content per skin organoid after stimulation with different concentrations of H_2_O_2_. Significance was analyzed by Student’s *t*-test. (D and E) Representative images (D) and quantitative analysis (E) of ROS staining in skin organoids after stimulation with different concentrations of H_2_O_2_. Significance was analyzed by Student’s *t*-test. Scale bar: 200 μm. (F) 3D heatmap comparing storage modulus (G’) and loss modulus (G’) of HAMA hydrogel vs Pt@HAMA nanohybrid hydrogels. (G) Schematic of dissolved oxygen (DO) measurement within hydrogels and culture medium. (H) DO differences within hydrogels and culture medium between the two systems. (I) Schematic illustrating the biological mechanism by which nanohybrid hydrogel culture enhances spheroid formation and protein expression.

To further evaluate the potential of the nano‑hybrid hydrogel constructed from hybrid units for future clinical applications, we established a human‑derived skin organoid model to assess its antioxidant protective function. The results demonstrated that the Pt@HAMA‑based nanohybrid hydrogel can effectively protect skin organoids from damage caused by exogenous ROS. High concentrations of H_2_O_2_ lead to the disintegration of skin organoids (Fig. 5A, left, as indicated by the yellow arrow). We quantified this phenomenon and found that the nanohybrid hydrogel constructed based on Pt@HAMA hybrid units significantly reduces the disintegration of skin organoids under high-concentration H_2_O_2_ stimulation (Fig. 5B). Meanwhile, we performed 3D GLO assays on skin organoids in different hydrogels (Fig. 5C). The ATP levels in skin organoids within the nanohybrid hydrogel groups under 1 mM and 10 mM H_2_O_2_ exposure were significantly higher, indicating a notable increase in the survival rate of skin organoids. This aligns with previous sequencing results, suggesting that the nanohybrid hydrogel not only reduces ROS levels but also effectively converts them into oxygen to support skin organoids. Furthermore, we assessed the ROS levels in the organoids. As the concentration of H_2_O_2_ stimulation increased, the accumulation of ROS in the organoids also rose. The results show that, starting from 1 mM H_2_O_2_, the Pt@HAMA-based nanohybrid hydrogel effectively reduces the accumulation of ROS in skin organoids (Fig. 5D and E). These results provide important experimental evidence for advancing the application of this material system in clinical directions such as regenerative medicine and refractory wound repair.

Based on the above results, we further characterized the static/dynamic mechanical properties of the two gels used for cell culture using a bio-nanoindenter. The results showed that Pt@HAMA in the nanohybrid hydrogel, acting as crosslinking points, effectively maintained the internal structure of the nanohybrid hydrogel during cell growth. Compared to the control hydrogel, its mechanical properties were closer to *in vivo* levels (Fig. 5F). The suitable viscoelasticity of the nanohybrid hydrogel promoted cells to form multicellular spheroids through proliferation and self-assembly. In contrast, both the storage modulus and loss modulus of the control hydrogel were significantly lower than *in vivo* levels (Fig. 5F), resulting in insufficient cell adhesion sites within the hydrogel. The weak cell adhesion ultimately caused cells to settle at the bottom of the culture dish and grow adherently (Fig. 4A). The mechanical differences between the two hydrogels are consistent with previous characterization results. Simultaneously, we characterized the dissolved oxygen levels inside both hydrogels and in the culture medium using a needle-type dissolved oxygen probe (Fig. 5G). The results demonstrated that the biocatalytic function provided by the nanohybrid gel effectively converted excess H_2_O_2_ produced by cells in the hydrogel culture system into oxygen. Compared to the control hydrogel, this significantly improved the overall dissolved oxygen level in the culture system (Fig. 5H). Combined with bioinformatic analysis, the results indicate that dissolved oxygen within the nanohybrid hydrogel can freely diffuse into cells, providing enhanced oxygen supply for mitochondrial ATP synthesis. In summary, the nanohybrid hydrogel promotes the formation of compact multicellular spheroids by 293T-PDL1(h) cells, enhances the endoplasmic reticulum’s capacity for folding and processing nascent proteins, and supplies oxygen through hybrid units, ultimately achieving highly efficient target protein expression (Fig. 5I).

## CONCLUSION

This study successfully developed a novel strategy for microscale organic-inorganic nanohybridization guided by natural biomacromolecules. By systematically analyzing the multilevel structure of hybrid units constructed through this strategy using a blob model, four core functionalities were systematically integrated. The constructed nanohybrid hydrogel based on hybrid units simultaneously achieves physical microenvironment simulation and adaptive biocatalytic functions, successfully promoting cellular self-organization and significantly improving hypoxic microenvironments without additional oxygen supply. Validation studies demonstrated that this hybrid material enhances mammalian cell production of PD-L1 protein by nearly 10-fold compared to conventional methods. Additionally, it exhibits significant application potential in cutting-edge fields such as hydrogel fiber weaving and cellular 3D printing, establishing a novel paradigm for the design of multifunctional biomimetic materials.

## Supplementary Material

nwag099_Supplemental_Files
