# Supplementary material for: A novel biomacromolecule-predominated hybrid unit: from design, characterization to application
Source: Natl Sci Rev. 2026 Feb 14;13(7):nwag099. doi: 10.1093/nsr/nwag099 (PMC13056720; doi:10.1093/nsr/nwag099)
Supplement: nwag099_Supplemental_Files [file nwag099_supplemental_files.zip › Supplementary data.pdf]

Supporting Information

A Novel Biomacromolecule-Predominated Hybrid Unit: From Design, Characterization to Application

Ke Hu (胡克)[1†], Ziying Zhou (周子莹)[2†], Zhaobin Guo (郭兆彬)[3], Hongxu Meng (孟泓旭)[4], Xuzhi Hu (胡徐智)[5], Jiamin Zhang (张佳敏)[1], Jiayi Zhu (朱嘉仪)[2], Ruichun Luo (罗瑞春)[2], Guoyin Chen (陈国印)[2*], Tingting Yu (俞婷婷)[6*], Meifang Zhu (朱美芳)[2*]

Materials and Methods

Materials

Methacrylated hyaluronic acid (HAMA) was purchased from Shanghai Yuju Technology Co., Ltd., methacrylated sodium alginate (AlgMA) was purchased from EFL-Tech (Suzhou) Co., Ltd., and methacrylated gelatin (GelMA) was purchased from Jiangyin Sisteyi Biotechnology Co., Ltd. Sodium borohydride ($NaBH_4$, ＞99%) was purchased from Sinopharm Chemical Reagent Co., Ltd. All other undeclared materials were sourced from Sigma-Aldrich Pte Ltd.

Cell line and cell culture

HEK-293T-PDL1(h)-Puro cells were purchased from GenScript Biotech Corporation (Nanjing, China, M00544). 293T-PDL1(h) cells were cultured in Dulbecco’s Modified Eagle Medium (Thermo Fisher Scientific, Waltham, MA, 12800017) containing 10% fetal bovine serum (Thermo Fisher Scientific, Waltham, MA, 10270-106), 2 mM L-glutamine (Thermo Fisher Scientific, Waltham, MA, 35050061), 1 mM Sodium Pyruvate (Thermo Fisher Scientific, Waltham, MA, 35050061) and 1×Penicillin, Streptomycin and Neomycin Antibiotic Mixture (Thermo Fisher Scientific, Waltham, MA, 15640055). HepG2 cells were purchased from Zhong Qiao Xin Zhou Biotechnology Co. Ltd (Shanghai, China) and cultured in Eagle's Minimum Essential Medium (Zhong Qiao Xin Zhou, ZQ-300) supplemented with 10% FBS (Gibco) and 1×Penicillin. HUVECs (Zhong Qiao Xin Zhou Biotechnology Co. Ltd) were cultured in Endothelial Cell Medium (ScienCell#1001)

Preparation and Characterization of Hybrid Units

Pt@HAMA, Pt@AlgMA and Pt@GelMA hybrid units： 50 mg each of HAMA, AlgMA, and GelMA was dissolved separately in 1 mL ultrapure water, then mixed with chloroplatinic acid aqueous solution (10 mg/mL, 1 mL). An aqueous solution of $NaBH_4$ was prepared (10 mg/mL, 10 mL) and maintained in an ice bath at 4°C. The mixture of biopolymer and chloroplatinic acid was added dropwise into the $NaBH_4$ solution at a rate of 24 mL/h under continuous ultrasonic irradiation (40 kHz, 0.5 W/cm^2) in an ice bath at 4°C for 30 min. Subsequently, the system was heated to 37°C and reacted for an additional 30 min. After completion, the product was dialyzed under neutral conditions for 3 days. Finally, the sample was lyophilized to obtain Pt@HAMA, Pt@AlgMA, and Pt@GelMA hybrid units.

Pt@HAMA-1, -2 and -3 hybrid units: 50 mg, 100 mg and 200 mg HAMA was dissolved separately in 1 mL ultrapure water, then mixed with chloroplatinic acid aqueous solution (10 mg/mL, 1 mL). An aqueous solution of $NaBH_4$ was prepared (10 mg/mL, 10 mL) and maintained in an ice bath at 4°C. The mixture of biopolymer and chloroplatinic acid was added dropwise into the $NaBH_4$ solution at a rate of 24 mL/h under continuous ultrasonic irradiation (40 kHz, 0.5 W/cm^2) in an ice bath at 4°C for 30 min. Subsequently, the system was heated to 37°C and reacted for an additional 30 min. After completion, the product was dialyzed under neutral conditions for 3 days. Finally, the sample was lyophilized to obtain Pt@HAMA-1, -2 and -3 hybrid units.

Various analytical techniques were employed to investigate the properties and microstructure and of the samples, including X-ray Photoelectron Spectroscopy (XPS) (Thermo Fisher ESCALAB 250Xi), Thermogravimetry Analysis (TG) (Netzsch STA 449 F3 Jupiter®), Fourier Transform Infrared Spectroscopy (FTIR) (Nicolet 380 FT-IR), Transmission Electron Microscope (TEM) (Thermo Fisher Talos F200X G2). Small-Angle X-ray Scattering (SAXS) measurements were performed at the Shanghai Synchrotron Radiation Facility (SSRF) beamline using a Pilatus 2M two-dimensional detector. Experimental parameters were set as follows: X-ray wavelength of 1.033 Å^{-1}, sample-to-detector distance of 2702 mm. Samples were dissolved in aqueous solution at a concentration of 3 mg/mL. Three exposures were collected for each sample, with 5 s exposure time per frame. The SAXS data were fitted using appropriate models by the SasView software version 6.0.1. Atomic Force Microscopy - Infrared Spectroscopy (AFM-IR) measurements were performed at the Nano Surfaces & Metrology Division of Bruker (Shanghai) using a Nano-IR3 system (Bruker Corporation, USA). Scanning was conducted in contact mode with gold-coated silicon probes (spring constant: 0.07-0.4 N/m,

Bruker Corporation, USA). Polarized IR laser wavelengths were set to specific wavenumbers for each hybrid material: Pt@HAMA: 1044 cm^{-1} and 1618 cm^{-1}; Pt@AlgMA: 1418 cm^{-1} and 1618 cm^{-1}; Pt@GelMA: 1548 cm^{-1} and 1656 cm^{-1}. Small-Angle Neutron Scattering (SANS) measurements were performed on SANS beamline at China Spallation Neutron Source (CSNS) using the same setup as reported previously [1]. Nanohybrid units were dissolved in D_2O at a concentration of 5 mM, loaded into 2 mm path-length quartz cells (Hellma GmbH, Type 120) and measured at 20°C. The SANS data were reduced using the Mantid framework and fitted using appropriate models by the SasView software version 6.1.0.

Enzyme-like Activity Characterization of Hybrid Units

Peroxidase-like Activity

For Pt@HAMA, Pt@AlgMA and Pt@GelMA hybrid units:

(1) Enzyme concentration dependence

Add 200 μL of acetate buffer (pH = 4.3), 10 μL of 10 mg/mL TMB, 20 μL of H_2O_2 solution (0.1 M), add 20 μL of hybrid units (Pt concentration: 12.5 μg/mL, 10 μg/mL,7.5 μg/mL, 5 μg/mL, 2.5 μg/mL, 1.25 μg/mL) respectively, transfer to the enzyme standard, shake rapidly for 2 s, read the absorbance at 650 nm, read every The absorbance at 650 nm was read every 10 s. The readings were taken 18 times. (n = 3 for each group)

(2) H_2O_2 concentration dependence

Add 200 μL acetate buffer (pH = 4.3), 10 μL 2.5 mg/mL TMB, 0.25, 0.5, 1, 2, 4, 6, 8 mM H_2O_2 solution, incubate at 30°C for 5 min, then add 20 μL hybrid units (Pt concentration: 12.5 μg/mL), mix well, transfer to the enzyme marker quickly, shake rapidly for 2 s, read every 10 s, and take 18 readings. The absorbance of the system at 650 nm was measured. The Mie kinetic parameters were calculated using the Mie equation. (n = 3 for each group)

(3) TMB concentration dependence

Add 200 μL of acetate buffer (pH = 4.3) and 20 μL of H_2O_2 solution (0.1 M) to the transparent ninety-six-well plate, add 0.005, 0.01, 0.02, 0.04, 0.08, 0.12, 0.17, 0.42 mM TMB solution respectively, incubate for 5 min at 30°C, then add 20 μL of hybrid units (Pt concentration: 12.5 μg/mL), mix well, transfer to the enzyme marker quickly and shake rapidly for 2 s. The absorbance of the system at 650 nm was detected by taking 18 readings every 10 s. The Mie kinetic parameters were calculated using the Mie equation. (n = 3 for each group)

(4) pH dependence

Add 200 μL of buffer (pH = 3.6, pH = 4.2, pH = 5.2, pH = 6.0, pH = 7.4) respectively and 20 μL of H_2O_2 solution (0.1 M) to the transparent ninety-six-well plate, add TMB solution (1

mg/mL), then add 20 μL of hybrid units (Pt concentration: 12.5 μg/mL), mix well, transfer to the enzyme marker quickly and shake rapidly for 2 s. The absorbance of the system at 650 nm was detected by taking 18 readings every 10 s. (n = 3 for each group)

For Pt@HAMA-1, -2 and -3 hybrid units:

(1) Enzyme concentration dependence

Add 200 μL of acetate buffer (pH = 4.3), 10 μL of 10 mg/mL TMB, 20 μL of H_2O_2 solution (0.1M), add 20 μL of hybrid units (Pt concentration: 12.5 μg/mL, 10 μg/mL, 7.5 μg/mL, 5 μg/mL, 2.5 μg/mL, 1.25 μg/mL) respectively, transfer to the enzyme standard, shake rapidly for 2 s, read the absorbance at 650 nm, read every The absorbance at 650 nm was read every 10 s. The readings were taken 18 times. (n = 3 for each group)

(2) H_2O_2 concentration dependence

Add 200 μL acetate buffer (pH = 4.3), 10 μL 2.5 mg/mL TMB, 0.25, 0.5, 1, 2, 4, 6, 8 mM H_2O_2 solution, incubate at 30°C for 5 min, then add 20 μL hybrid units (Pt concentration: 12.5 μg/mL), mix well, transfer to the enzyme marker quickly, shake rapidly for 2 s, read every 10 s, and take 18 readings. The absorbance of the system at 650 nm was measured. The Mie kinetic parameters were calculated using the Mie equation. (n = 3 for each group)

(3) TMB concentration dependence

Add 200 μL of acetate buffer (pH = 4.3) and 20 μL of H_2O_2 solution (0.1 M) to the transparent ninety-six-well plate, add 0.005, 0.01, 0.02, 0.04, 0.08, 0.12, 0.17, 0.42 mM TMB solution respectively, incubate for 5 min at 30°C, then add 20 μL of hybrid units (Pt concentration: 12.5 μg/mL), mix well, transfer to the enzyme marker quickly and shake rapidly for 2 s. The absorbance of the system at 650 nm was detected by taking 18 readings every 10 s. The Mie kinetic parameters were calculated using the Mie equation. (n = 3 for each group)

(4) pH dependence

Add 200 μL of buffer (pH = 3.5, pH = 3.7, pH = 4.0, pH = 4.3, pH = 4.6, pH = 5.0, pH = 6.0, pH = 5.3, pH = 5.6, pH = 6.0, pH = 6.3, pH = 6.6, pH = 7.0, pH = 7.3, pH = 7.6, pH = 8.0) respectively and 20 μL of H_2O_2 solution (0.1 M) to the transparent ninety-six-well plate, add TMB solution (1 mg/mL), then add 20 μL of hybrid units (Pt concentration: 12.5 μg/mL), mix well, transfer to the enzyme marker quickly and shake rapidly for 2 s. The absorbance of the system at 650 nm was detected by taking 18 readings every 10 s. (n = 3 for each group)

Catalase-like Activity

For Pt@HAMA, Pt@AlgMA and Pt@GelMA hybrid units:

Preparation of 200 μL buffer solutions at different pH values (pH = 4.2, 5.2, 6.0, 7.4, 8.23), followed by addition of 20 μL hybrid units (25 μg/mL) and 30 μL H_2O_2 solution (5 M). Dissolved oxygen changes in the system were measured using a dissolved oxygen meter (Presens), with triplicate experimental runs. The initial reaction velocity V_0 was then determined by fitting the oxygen concentration versus time curve.

For Pt@HAMA-1, -2 and -3 hybrid units:
Preparation of 200 μL buffer solution (pH = 8.23), addition of 20 μL hybrid units (25 μg/mL) and 30 μL H_2O_2 solution (5 M), measurement of dissolved oxygen changes in the system using a dissolved oxygen meter (Presens). The experiment was repeated three times. The initial reaction velocity V_0 was then determined by fitting the oxygen concentration versus time curve.

Preparation and Characterization of Nanohybrid Hydrogel

For Enzyme-like Activity Characterization
The nanohybrid hydrogel was synthesized by dissolving GelMA (4% w/w, 50% grafting degree) and hybrid units (0.05% w/w) in 1 mL ultrapure water, followed by addition of Lithium phenyl-2,4,6-trimethylbenzoylphosphinate (LAP) (0.5% w/w) and 405 nm photopolymerization in cell culture plate (96-well) for 1 min.
Peroxidase-like Activity: the chromogenic reaction was initiated by adding 100 μL acetate buffer (pH = 4.0) and 20 μL H_2O_2 (10 M) into nanohybrid hydrogel-immobilized cell culture plates (96-well). After 10 μL TMB (10 mg/mL) injection, time-dependent blue coloration was documented with photographic monitoring.
Catalase-like Activity: bubble evolution kinetics of nanohybrid hydrogel were monitored following addition of 100 μL buffer (pH = 8.2) and 30 μL H_2O_2 (10 M) to 96-well plates immobilized with nanohybrid hydrogels.

For Dynamic and Static Mechanical Properties Characterization
The nanohybrid hydrogel was synthesized by dissolving GelMA (3% w/w, 50% grafting degree) and hybrid units (0.1% w/w) in 1 mL ultrapure water, followed by addition of Lithium phenyl-2,4,6-trimethylbenzoylphosphinate (LAP) (0.5% w/w) and 405 nm photopolymerization in 35 mm confocal dish for 0.5 min.
The sample was subjected to static mechanical analysis and DMA-based dynamic mechanical analysis using a Piuma Bio-nanoindenter (Optics11) in displacement control mode. The nanoindenter optical probe was spherical with a stiffness of 0.52 N m^{-1} and a tip radius of 24.5

μm. For the static mechanical analysis, the indentation was performed to a depth of 20 μm at a speed of 10 μm/s and unloaded. For the DMA-based dynamic mechanical analysis, the indentation was performed to a depth of 20 μm at a speed of 10 μm/s, followed by a 20 s hold period at maximum depth during which a 10 Hz sinusoidal oscillation with a uniform amplitude of 200 nm was axially superimposed; a 2 s relaxation interval was subsequently implemented after oscillation at each tested frequency.

Preparation and Characterization of Nanohybrid Hydrogel Fiber

The hydrogel optical fiber fabrication followed our previously reported method [4]. Specifically: The core solution was prepared by dissolving 0.015 g I2959, 0.045 g Poly(ethylene glycol) diacrylate (PEGDA, MW: 250), and 2.955 g acrylamide in 10 mL ultrapure water. The cortex solution contained 0.01 g Pt@HAMA, 0.015 g PEGDA (MW: 250), 0.985 g acrylamide, and 0.005 g I2959 in 10 mL sodium alginate solution (2 wt.%). Fiber spinning was conducted using a 2 wt.% calcium chloride coagulation bath with a 21-gauge needle, employing extrusion rates of 2.96 mL/h for the cladding layer and 1.78 mL/h for the core layer.

Peroxidase-like Activity: the chromogenic reaction was initiated by adding 100 μL acetate buffer (pH= 4.0) and 300 μL H_2O_2 (10 M) into a petri dish. Hydrogel fiber segments (2 cm length) were immersed and blue coloration development documented visually.
Catalase-like Activity: bubble evolution was initiated by adding 100 μL buffer (pH = 8.0) and 300 μL H_2O_2 (10 M) into a petri dish. Hydrogel fiber segments (2 cm length) were immersed and bubble evolution development documented visually.

Bioprinting

A hydrogel precursor solution was prepared by dissolving 2% w/v hyaluronic acid methacrylate (HAMA; Cyberediad, YJPH03-2G) and 0.5% w/v lithium phenyl-2,4,6-trimethylbenzoylphosphinate (LAP; Sigma, 900889) in phosphate-buffered saline (PBS; ThermoFisher, 10010023). For one experimental group, 0.1% w/v Pt@HAMA was incorporated. The solution was incubated at 57°C for approximately 30 minutes with bath sonication to ensure complete dissolution, followed by sterile filtration through a 0.22 μm membrane (Beyotime, FF372-10pcs). HepG2 hepatocytes and human umbilical vein endothelial cells (HUVECs; Shanghai Zhong Qiao Xin Zhou Biotechnology Co. Ltd) were centrifuged and resuspended in their respective precursor solutions to final densities of 2×10^7 cells/mL and 1×10^7 cells/mL. By using a digital light processing (DLP) bioprinter, HepG2-

laden hydrogel was patterned into hexagonal hepatic lobule-like constructs (300 μm height) under 40% light intensity with 8 s exposures per layer. Uncrosslinked residual bioink and cells were removed by washing twice with PBS. Subsequently, HUVEC-laden bioink was printed into channel-like structures (300 μm height) within the HepG2 framework. After two additional PBS washes, constructs were cultured in a 1:1 mixture of EMEM (Gibco) and Endothelial Cell Medium (ScienCell) at 37°C under 5% CO_2.

Hydrogen Peroxide Oxidative Stress Assay: Monocultured HepG2 constructs were bioprinted as described. Following 48 hours of culture adaptation, half of the samples were exposed to 500 μM H_2O_2 (Sigma, 323381) for 24 hours. Viability was assessed using a dual-fluorescence live/dead assay: A working solution containing 5 μM Calcein-AM and 15 μM propidium iodide (PI) was prepared in 1 × Assay Buffer (Yeasen, 40747ES76) from 2 mM and 1.5 mM stock solutions, respectively. After removing culture supernatant and washing twice with PBS, 200 μL of the working solution was added per well and incubated at 37°C for 15 minutes. Following two final PBS washes, cell viability was quantified *via* fluorescence microscopy.

3D Cell culture

GelMA (3% w/w) and Pt@HAMA (0.1% w/w for nanohybrid hydrogel) or HAMA (0.1% w/w for control hydrogel) were dissolved in PBS with MA-RGD (0.5% w/w) and LAP (0.5% w/w). Stir in a light-protected heating bath for 1 h until formation of a transparent hydrogel precursor, followed by sterile filtration through a 0.22 μm filter. The precursor was mixed with cell resuspension, seeded 1600 μL per well (containing 1.6×10^5 293T-PDL1 cells) in 6-well plates and photocrosslinked *via* 405 nm irradiation (30 s) to form hydrogels. 2 mL fresh medium was added per well and the cells were incubated for subsequent observation and charaterization.

Immunofluorescence (IF) Staining

The culture medium was aspirated and the cells were gently rinsed once with PBS. Fixation was performed with 2% PFA for 15 min under appropriate safety precautions. Residual PFA was neutralized by washing three times with PBS-glycine solution. Cells were permeabilized using IF-Wash buffer for 20 min, followed by three gentle washes with IF-Wash buffer (5 min/wash with shaking). Blocking solution (2% normal goat serum) was added and incubated at room temperature for 1 h to minimize nonspecific binding. After aspiration of the blocking solution, primary antibody (1 : 250 dilution in IF-Wash buffer) was applied and incubated at 4°C for 48 h to ensure sufficient antibody binding. The primary antibody mixture was then

aspirated and unbound antibodies were removed through three washes with IF-Wash buffer (gentle shaking). Fluorescently-labeled secondary antibody (1 : 500 in IF-Wash buffer) and DAPI (for nuclear staining) were subsequently added, followed by 24 h incubation at 4°C. Finally, four stringent washes with IF-Wash buffer (gentle shaking, 10 min/wash) were conducted to eliminate unbound secondary antibodies.

Western Blot and Antibodies

Cells from both nanohybrid hydrogel and control hydrogel groups were collected, followed by protein extraction using RIPA lysis buffer and quantification *via* BCA assay. Subsequently, protein separation was performed through SDS-PAGE electrophoresis, after which proteins were transferred onto PVDF membranes. The membranes were incubated with primary antibodies (targeting specific proteins and internal controls) and HRP-conjugated secondary antibodies. Finally, protein bands were visualized using ECL substrate, and images were captured with a Western Blot imaging system. Quantitative analysis of band intensity was conducted using image processing software, whereby gray value ratios of target proteins to internal reference proteins were calculated. Statistical significance of differential protein expression across treatment groups was determined through rigorous analysis. The following antibodies were used: anti-GAPDH (Proteintech, Cat No.60004-1-Ig, CloneNo.1E6D9, 1:5000), anti-PDL-1 (Cell Signaling Technology, Cat No.13684T,1:1000), anti-TPR (Abcam, Cat No.ab170940, Clone No.EPR8982, 1:1000), anti-HSPA5 (Proteintech, Cat No.11587-1-AP,1:2000), anti-Sec62 (Abcam, Cat No.ab140644, Clone No.EPR9213,1:1000), anti-PDIA6 (Abcam, Cat No.ab154820,Clone No.EPR10132(B),1:1000).

High-throughput Reference-based mRNA-seq

Cells from nanohybrid hydrogel and control hydrogel groups were collected, followed by total RNA extraction using the RNeasy kit (Qiagen) according to the manufacturer's standardized protocol. The obtained RNA underwent quantitative quality control validation prior to subsequent library construction. Sequencing was performed on an Illumina platform, after which raw data were aligned and normalized. Gene expression values (counts and FPKM) were calculated, and differential expression analysis with statistical significance determination was conducted using the edgeR package in R based on predefined grouping criteria.

Protein Mass Spectrometry Analysis

Cells from nanohybrid hydrogel and control hydrogel groups were collected and total proteins were extracted. After peptide digestion, the samples were subjected to mass spectrometry analysis using LC-MS/MS. Subsequently, the DIA-NN software was employed for database searching, followed by bioinformatics analysis of the search results, which primarily included identification analysis, differential expression analysis, and functional analysis. After obtaining differentially expressed mRNAs and proteins, the R package was used to perform GO and pathway analyses on the identified differential genes.

Proteomics and Transcriptomics Analysis

Proteomic analysis was performed on multicellular spheroids by extracting total proteins, digesting them with trypsin, and analyzing the resulting peptides using liquid chromatography-tandem mass spectrometry (LC-MS/MS). Data were processed with MaxQuant and analyzed for differentially expressed proteins.

Gene Set Enrichment Analysis (GSEA) was conducted to identify enriched pathways, and protein-protein interaction (PPI) networks were constructed using the STRING database and visualized with Cytoscape.

For transcriptomic analysis, RNA was extracted from the same samples using the RNeasy Mini Kit (Qiagen), followed by RNA-seq on the Illumina NovaSeq 6000 platform. Raw data were aligned to the human reference genome (GRCh38) using STAR and analyzed for differential gene expression using DESeq2. Enrichment analyses for Gene Ontology (GO) and KEGG pathways, as well as GSEA, were performed to identify significant biological processes.

Statistical Analyses

Statistical analyses were performed with GraphPad Prism 8.0 (RRID:SCR_002798). Each measurement was repeated at least three times. Comparisons between indicated groups were performed using independent samples t-test. P values < 0.05 were considered statistically significant. *$P < 0.05$, **$P < 0.01$, and ***$P < 0.001$. n.s. not significant.

Skin Organoid Construction, 3D Culture and Antioxidant Assessment

Skin Organoid Construction: Skin organoids were formed by mixing HaCaT (HUM-iCell-s002) and Hdfs (HUM-iCELL-s001) cells at a 2:1 ratio, seeding 9,000 cells per well in ultra-low attachment 96-well U-bottom plates (Corning, 7007), and culturing for 3 days. The skin organoid culture medium was prepared by mixing HaCaT-specific medium (iCell-h066-001b) and Hdfs-specific medium (iCell-s001-002h) at a 2:1 ratio.

Skin Organoid 3D Culture: GelMA (3% w/w) and Pt@HAMA (0.1% w/w for nanohybrid hydrogel) or HAMA (0.1% w/w for control hydrogel) were dissolved in PBS with MA-RGD (0.5% w/w) and LAP (0.5% w/w). Stir in a light-protected heating bath for 1 h until formation of a transparent hydrogel precursor, followed by sterile filtration through a 0.22 μm filter. For 96-well plates, 100 μL of precursor was added per well; for 24-well plates, 400 μL of precursor was added per well. The precursor was mixed with organoids and photocrosslinked *via* 405 nm irradiation (30 s) to form hydrogels. After an 8 h adaptation culture, the organoids were treated with different concentrations of H_2O_2 for 48 h.

ATP Level Detection: For hydrogels in 96-well plates, 100 μL of CellTiter-Glo® 3D Reagent was added to each well and mixed thoroughly for 5 min to lyse the cells. The plate was then incubated at room temperature for an additional 25 min to stabilize the luminescence signal. Luminescence was recorded at 560 nm using a microplate reader.

ROS Level Detection: For hydrogels in 24-well plates, 500 μL of ROS staining solution (MCE, HY-D0940) diluted 1:1000 was added to each well and incubated at 37°C for 30 minutes, followed by imaging under a microscope.

Dissolved Oxygen Measurement in Hydrogel/in Culture Medium

The dissolved oxygen probe was inserted into the hydrogel to measure the dissolved oxygen content. After 20 seconds, the probe was raised into the culture medium, and the dissolved oxygen content in the culture medium was recorded after readings stabilized.

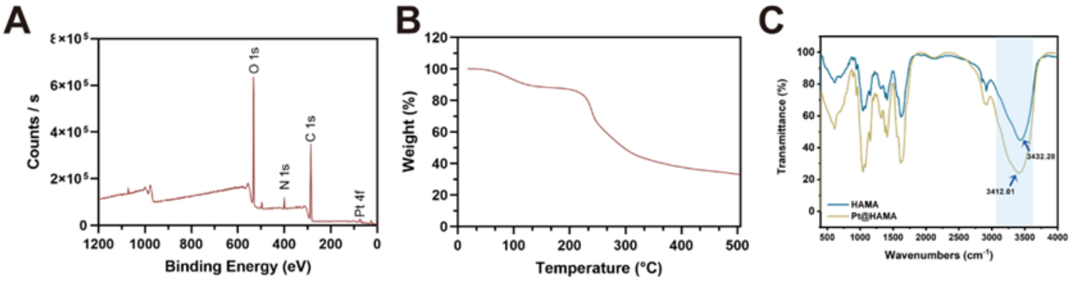

Figure S1. XPS(A), TGA(B)and FTIR(C) spectrum of Pt@HAMA.

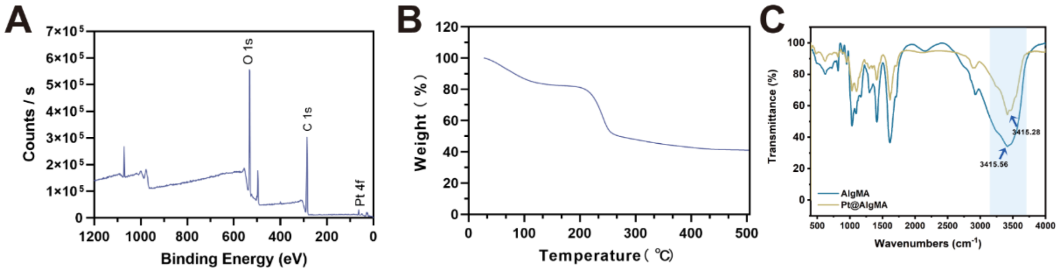

Figure S2. XPS(A), TGA(B)and FTIR(C) spectrum of Pt@AlgMA.

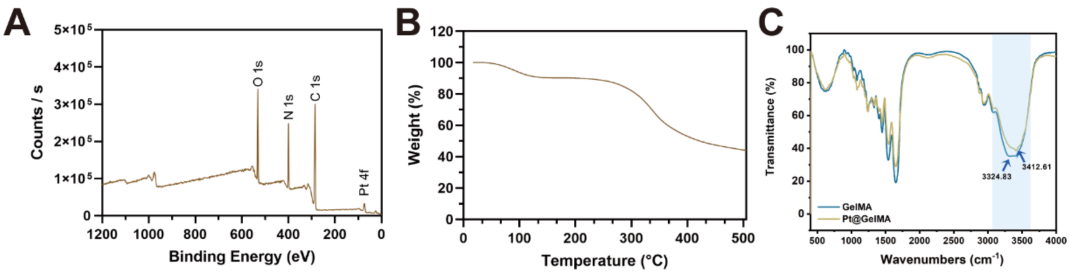

Figure S3. XPS(A), TGA(B)and FTIR(C) spectrum of Pt@GelMA.

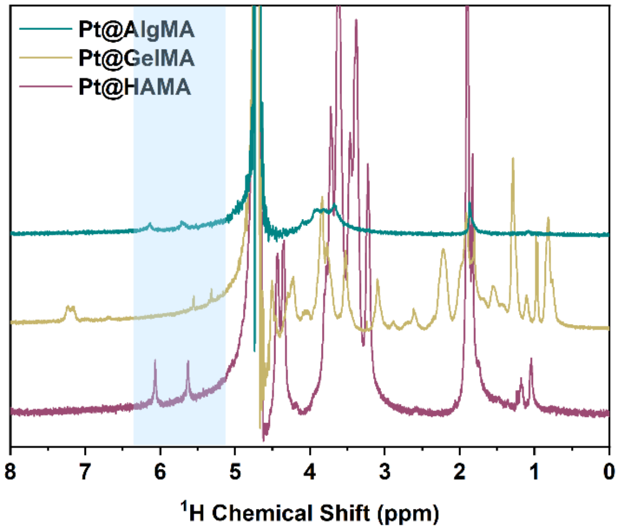

Figure S4. NMR spectrum of Pt@HAMA, Pt@AlgMA and Pt@GelMA.

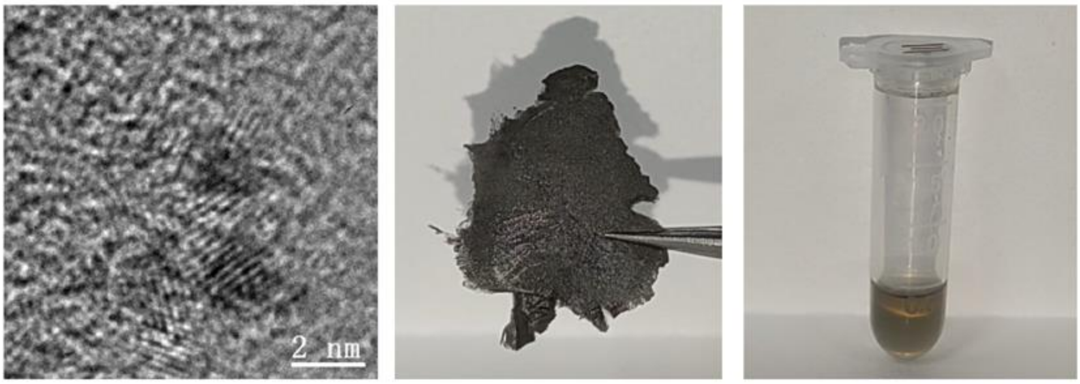

Figure S5. High-resolution TEM image (left), photograph of the Pt@HAMA sample after lyophilization (center), and redissolved sample solution in water (right).

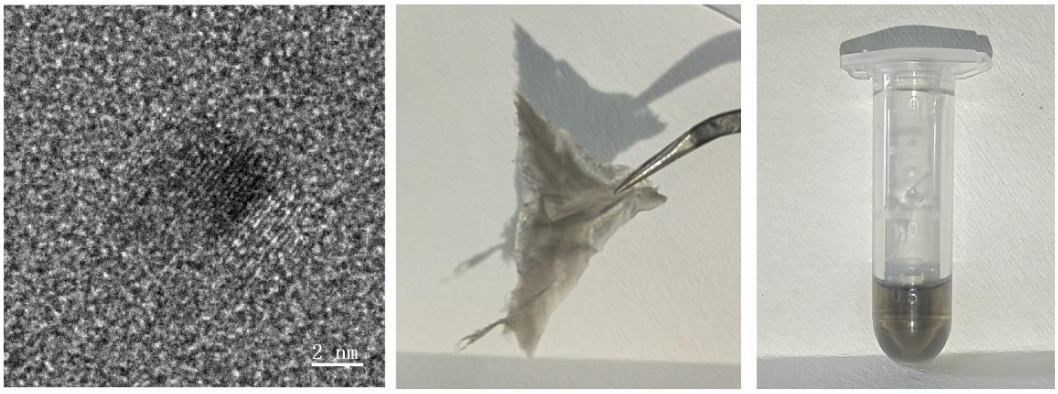

Figure S6. High-resolution TEM image (left), photograph of the Pt@HAMA sample after lyophilization (center), and redissolved sample solution in water (right).

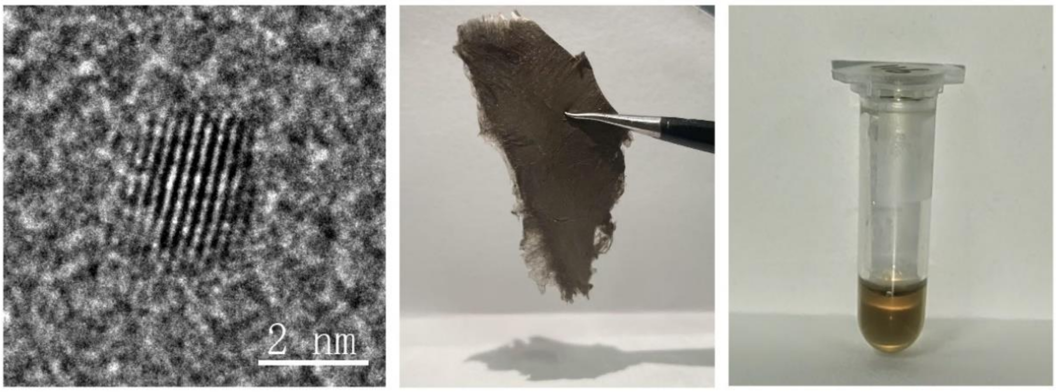

Figure S7. High-resolution TEM image (left), photograph of the Pt@HAMA sample after lyophilization (center), and redissolved sample solution in water (right).

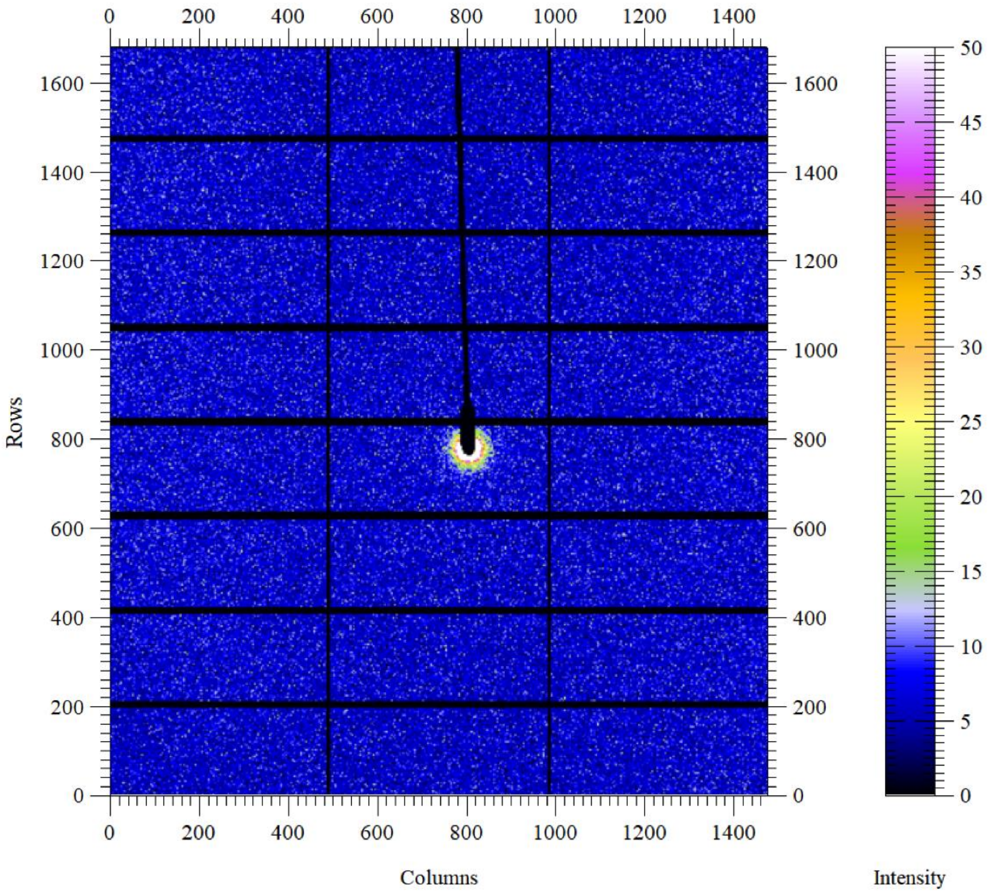

Figure S8. 2D SAXS image of water.

Table S1. Synchrotron radiation SAXS fitting parameters for hybrid units

	**HAMA**	**Pt@HAMA**	**AlgMA**	**Pt@AlgMA**	**GelMA**	**Pt@GelMA**
l(nm)	0.80	2.82	1.84	0.80	0.94	1.34
d_f	1.80	2.25	1.91	2.91	1.88	1.20
ξ(nm)	2.27	12.71	3.51	7.79	5.53	6.63
N_{blob}	7	30	3	752	28	7

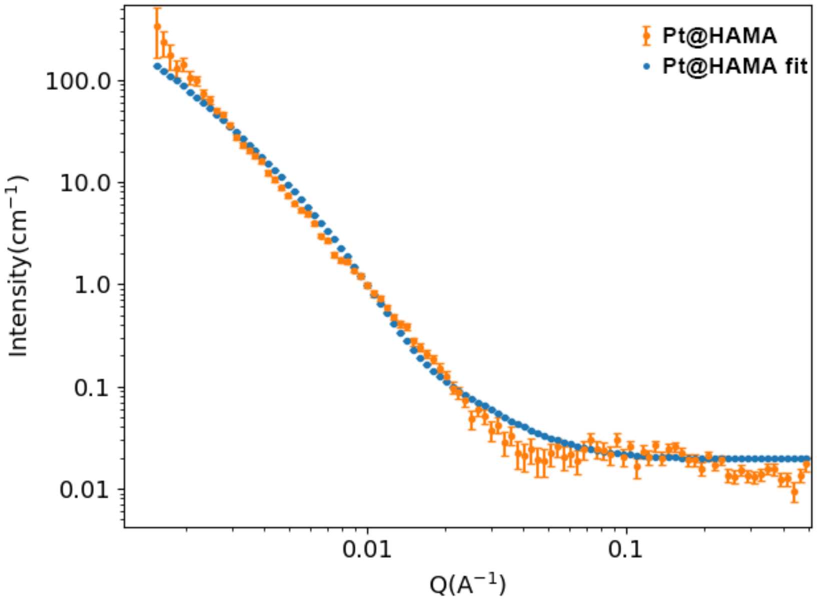

Figure S9. Small-Angle Neutron Scattering (SANS) pattern of Pt@HAMA.

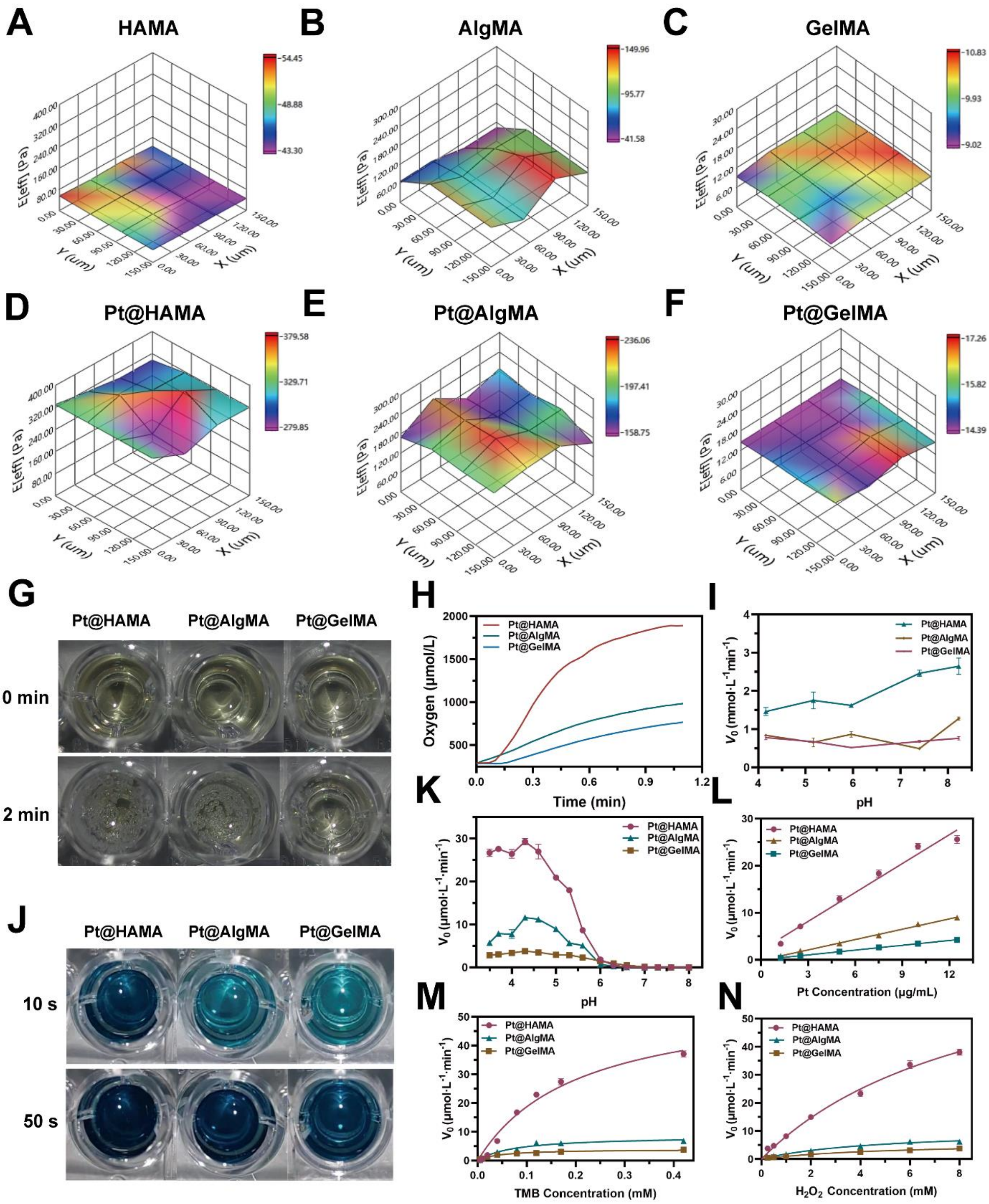

Figure S10. Distinct differences exist among hybrid units in enhancing hydrogel mechanical properties and biocatalytic activity. A-F: 3D heatmaps of Young's modulus for hydrogels prepared using: HAMA(A), AlgMA (B), GelMA(C), Pt@HAMA(D), Pt@AlgMA(E), Pt@GelMA(F) as building blocks. G: CAT-like enzyme catalytic activity of hydrogels prepared with hybrid units. H: Dissolved oxygen curves for the CAT-like enzyme reaction of hybrid units. I: pH dependence of CAT-like enzyme activity for hybrid units. J: POD-like enzyme

catalytic activity of hydrogels prepared with hybrid units. K: pH dependence of POD-like enzyme activity for hybrid units. L: Concentration dependence (hybrid units) for POD-like enzyme activity. M: TMB substrate concentration dependence for POD-like enzyme activity of hybrid units. N: H_2O_2 substrate concentration dependence for POD-like enzyme activity of hybrid units.

Table S2. Michaelis-Menten Kinetic Parameters of Hybrid Unit-Based POD-like Enzymes

		Pt@HAMA	Pt@AlgMA	Pt@GelMA
TMB	V_{max}(μmol/L/min)	57	8.485	3.992
	K_m(mM)	0.2048	0.07571	0.04668
H_2O_2	V_{max}(μmol/L/min)	82.26	10.38	6.126
	K_m (mM)	9.18	4.761	5.354

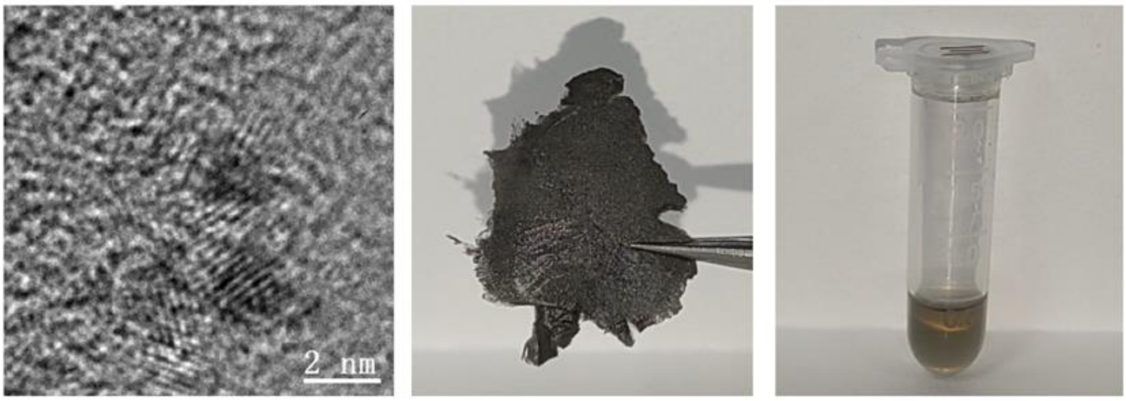

Figure S11. High-resolution TEM image (left), photograph of the Pt@HAMA-1 sample after lyophilization (center), and redissolved sample solution in water (right).

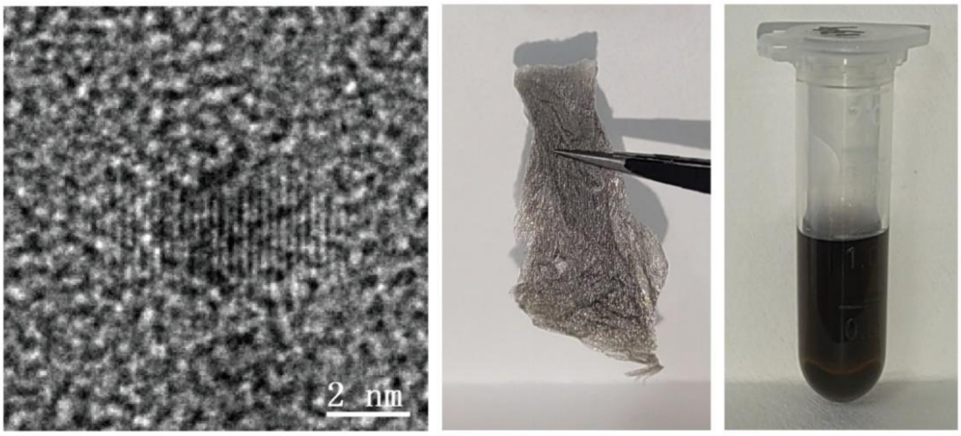

Figure S12. High-resolution TEM image (left), photograph of the Pt@HAMA-2 sample after lyophilization (center), and redissolved sample solution in water (right).

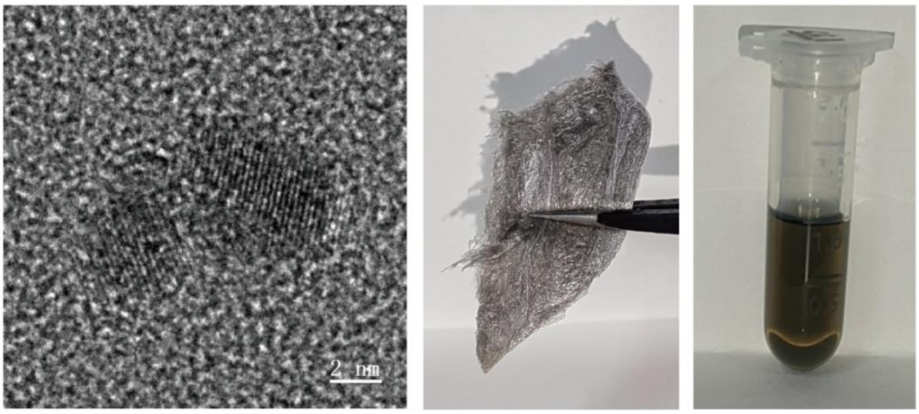

Figure S13. High-resolution TEM image (left), photograph of the Pt@HAMA-3 sample after lyophilization (center), and redissolved sample solution in water (right).

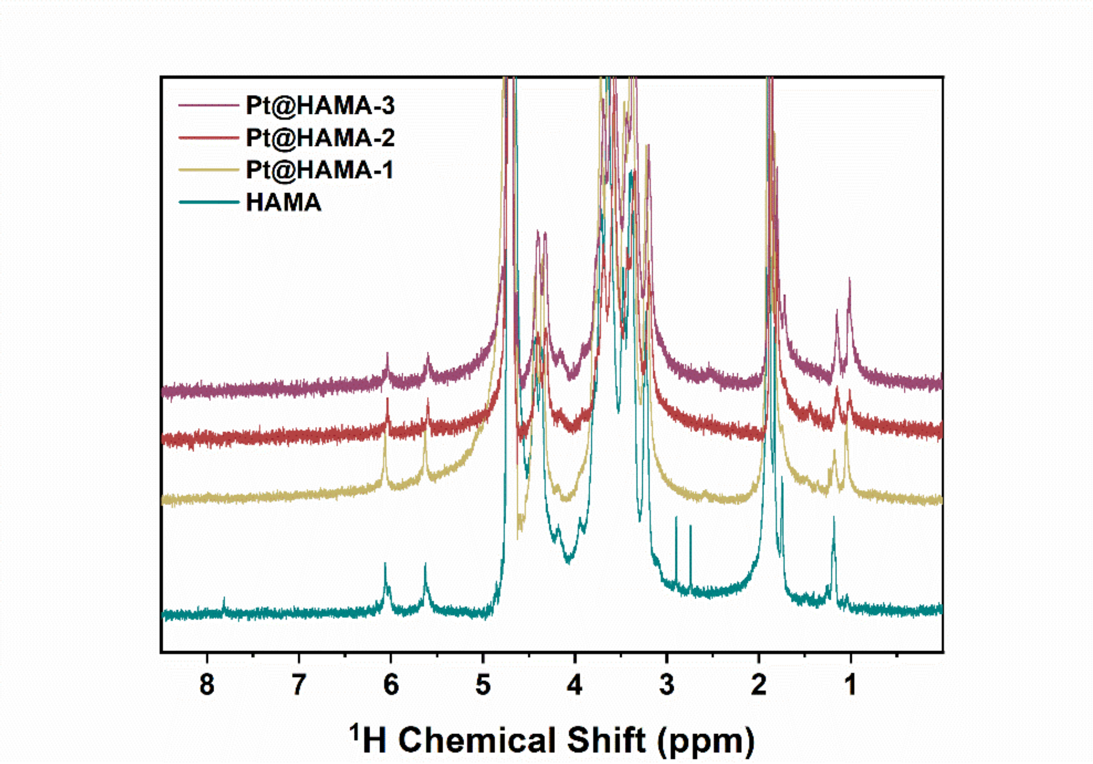

Figure S14. NMR spectrum of Pt@HAMA-1 , -2 and -3.

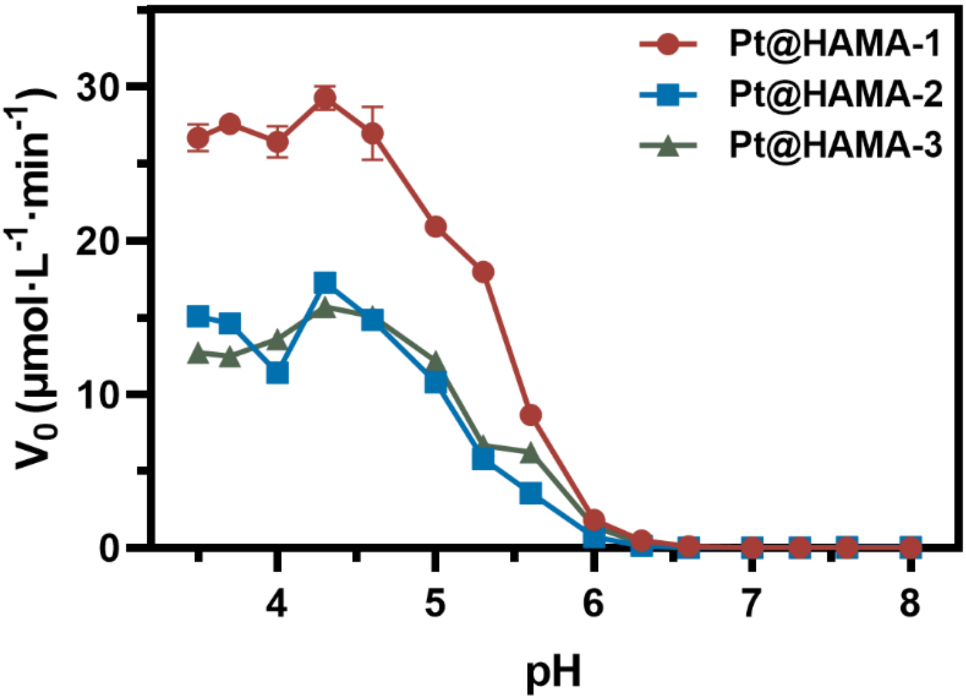

Figure S15. pH-Dependence of POD-like Enzymes of Pt@HAMA-1 , -2 and -3.

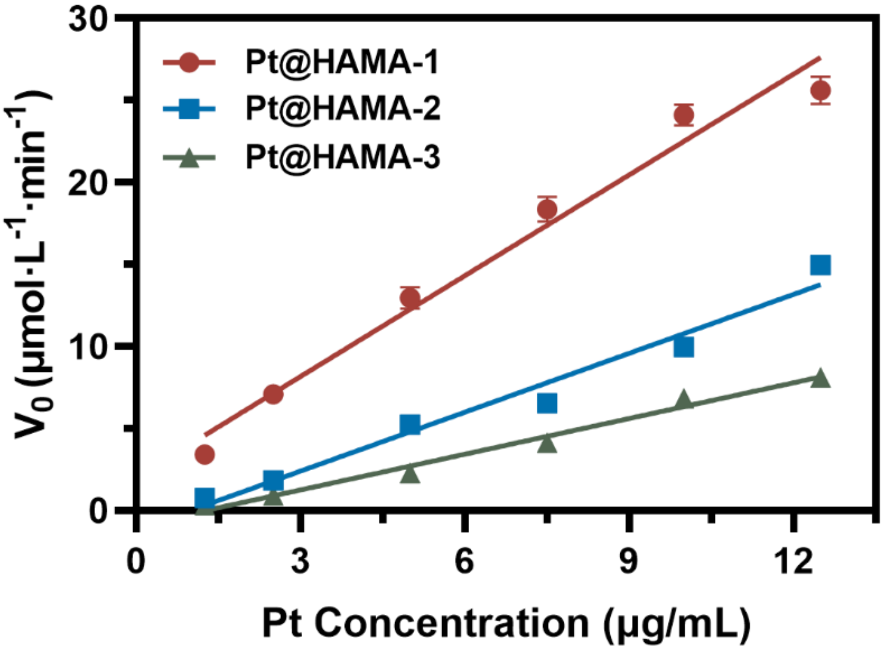

Figure S16. Concentration-Dependent of POD-like Enzymes of Pt@HAMA-1 , -2 and -3.

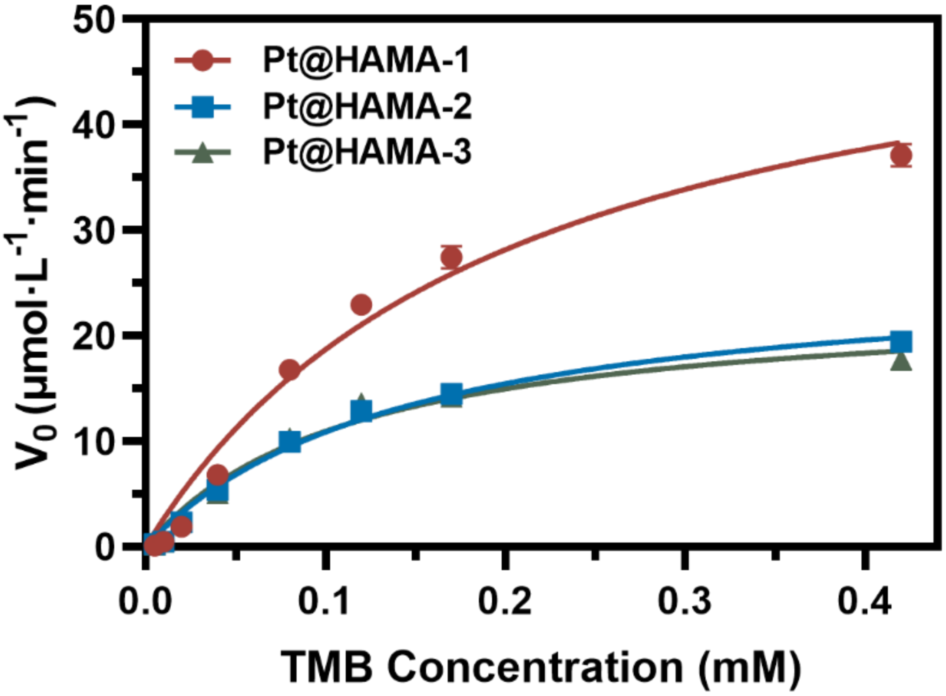

Figure S17. Concentration-Dependence of TMB Substrate for POD-like Enzymes of Pt@HAMA-1 , -2 and -3.

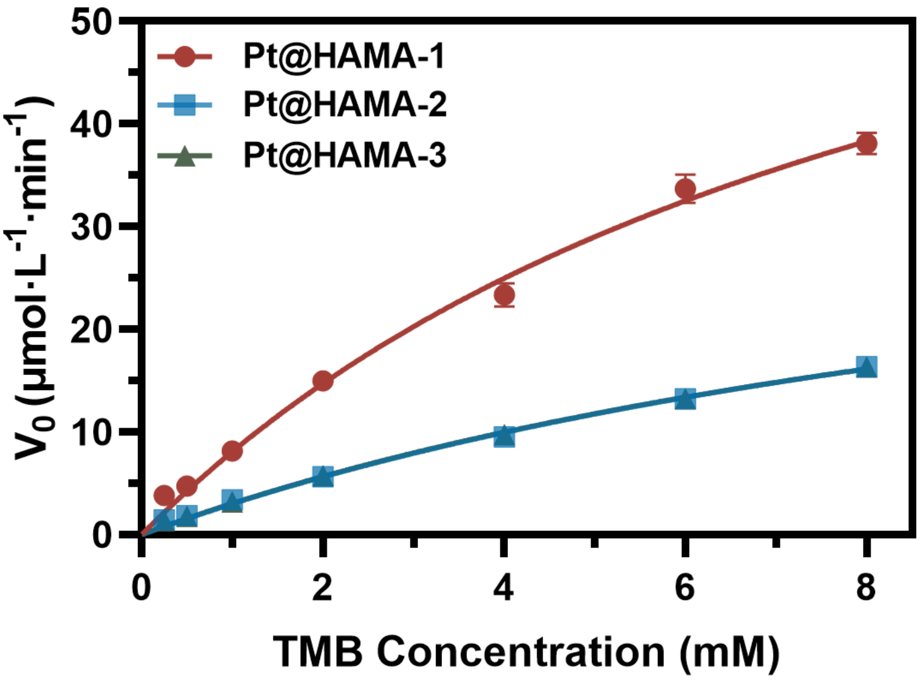

Figure S18. Concentration-Dependence of H_2O_2 Substrate for POD-like Enzymes of Pt@HAMA-1 , -2 and -3.

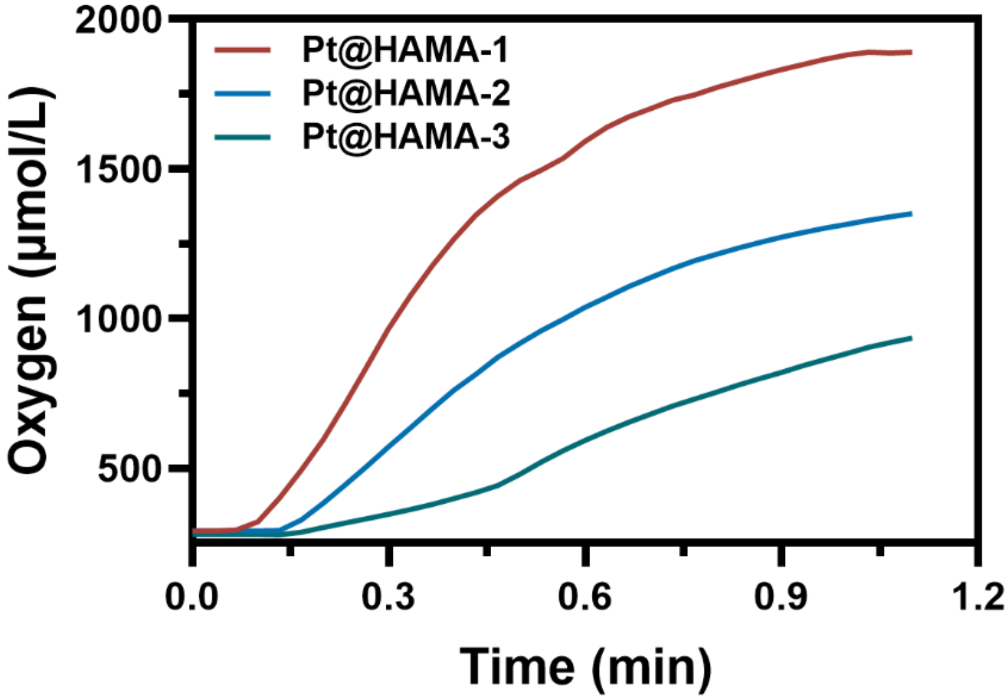

Figure S19. Dissolved Oxygen During the Reaction of CAT-like Enzymes of Pt@HAMA-1 , -2 and -3.

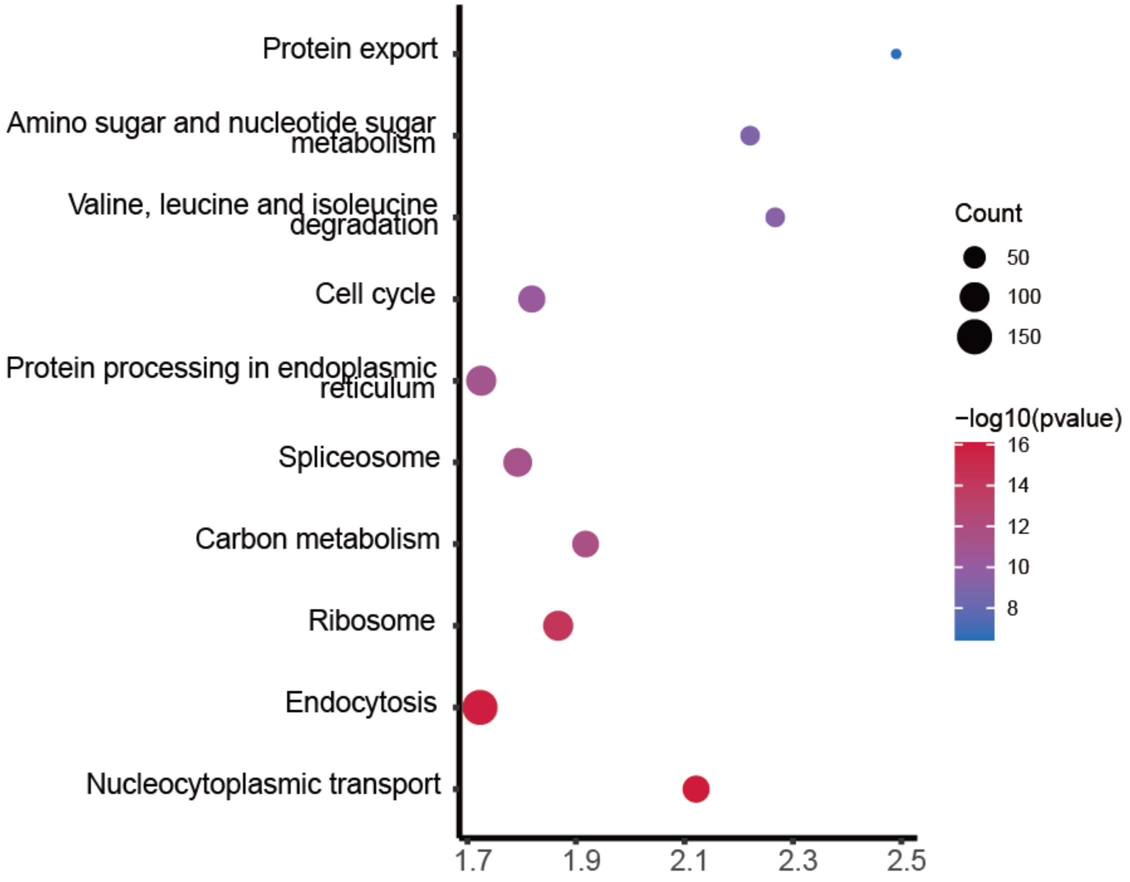

Figure S20. KEGG pathway enrichment analysis of differentially expressed proteins.

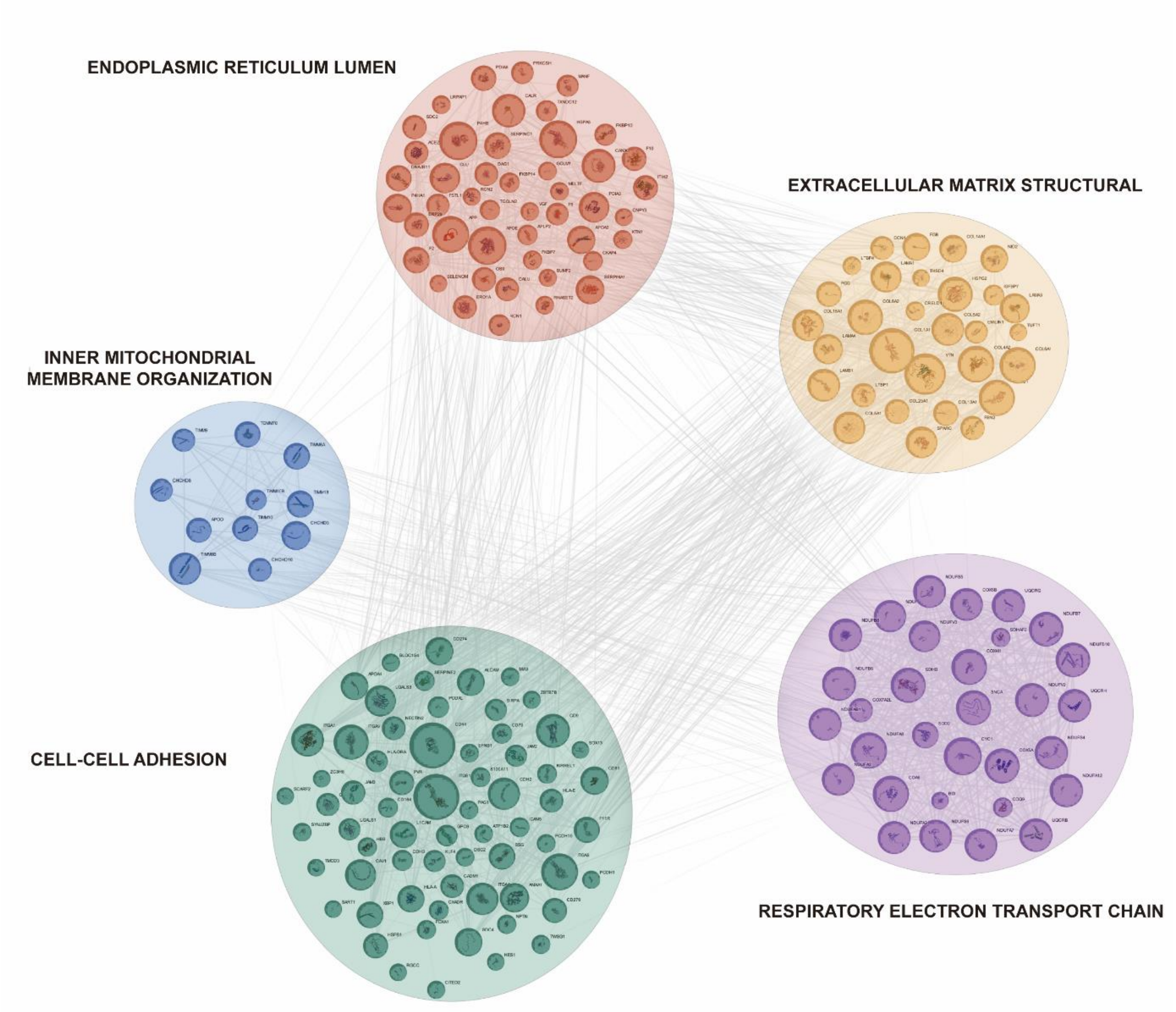

Figure S21. Protein-protein interaction networks of identified upregulated pathways in hydrogel-cultured spheroids.

Video S1. Hybrid hydrogel CAT-like catalytic reaction

Video S2. Hybrid hydrogel POD-like catalytic reaction

Video S3. CAT-like catalytic activity of 3D-printed hybrid hydrogel with logo‘MengTai‘

References

[1] X. Hu, M. Liao, K. Ding, J. Wang, H. Xu, K. Tao, F. Zhou, J.R. Lu, Neutron reflection and scattering in characterising peptide assemblies, Advances in Colloid and Interface Science (2023) 103033.

[2] Chen, G.; Hou, K.; Yu, N.; Wei, P.; Chen, T.; Zhang, C.; Wang, S.; Liu, H.; Cao, R.; Zhu, L.; Hsiao, B. S.; Zhu, M., Temperature-adaptive hydrogel optical waveguide with soft tissue-affinity for thermal regulated interventional photomedicine. Nature Communications 2022, 13 (1), 7789.
